# The role of small proteins in *Burkholderia cenocepacia* J2315 biofilm formation, persistence and intracellular growth

**DOI:** 10.1016/j.bioflm.2019.100001

**Published:** 2019-06-15

**Authors:** Heleen Van Acker, Aurélie Crabbé, Dukas Jurėnas, Lisa Ostyn, Andrea Sass, Simon Daled, Maarten Dhaenens, Dieter Deforce, Eline Teirlinck, Herlinde De Keersmaecker, Kevin Braeckmans, Laurence Van Melderen, Tom Coenye

**Affiliations:** a Laboratory of Pharmaceutical Microbiology, Ghent University, Ghent, Belgium; b Cellular and Molecular Microbiology, Université Libre de Bruxelles, Gosselies, Belgium; c Laboratory of Pharmaceutical Biotechnology, Ghent University, Ghent, Belgium; d Laboratory for General Biochemistry and Physical Pharmacy, Ghent University, Ghent, Belgium

**Keywords:** Small proteins, *Burkholderia*, Biofilm, Persistence

## Abstract

*Burkholderia cenocepacia* infections are difficult to treat due to resistance, biofilm formation and persistence. *B. cenocepacia* strain J2315 has a large multi-replicon genome (8.06 Mb) and the function of a large fraction of (conserved) hypothetical genes remains elusive. The goal of the present study is to elucidate the role of small proteins in *B. cenocepacia*, focusing on genes smaller than 300 base pairs of which the function is unknown. Almost 10% (572) of the *B. cenocepacia* J2315 genes are smaller than 300 base pairs and more than half of these are annotated as coding for hypothetical proteins. For 234 of them no similarity could be found with non-hypothetical genes in other bacteria using BLAST. Using available RNA sequencing data obtained from biofilms, a list of 27 highly expressed *B. cenocepacia* J2315 genes coding for small proteins was compiled. For nine of them expression in biofilms was also confirmed using LC-MS based proteomics and/or expression was confirmed using eGFP translational fusions. Overexpression of two of these genes negatively impacted growth, whereas for four others overexpression led to an increase in biofilm biomass. Overexpression did not have an influence on the MIC for tobramycin, ciprofloxacin or meropenem but for five small protein encoding genes, overexpression had an effect on the number of persister cells in biofilms. While there were no significant differences in adherence to and invasion of A549 epithelial cells between the overexpression mutants and the WT, significant differences were observed in intracellular growth/survival. Finally, the small protein BCAM0271 was identified as an antitoxin belonging to a toxin-antitoxin module. The toxin was found to encode a tRNA acetylase that inhibits translation. In conclusion, our results confirm that small proteins are present in the genome of *B. cenocepacia* J2315 and indicate that they are involved in various biological processes, including biofilm formation, persistence and intracellular growth.

## 1 Introduction

*Burkholderia cenocepacia* J2315 is a member of the *Burkholderia cepacia* complex (*Bcc*), a group of opportunistic pathogens that can cause severe lung infections in cystic fibrosis (CF) patients [1,2]. Infections are often difficult to treat due to resistance, biofilm formation and persistence [3]. Biofilms consist of aggregated cells embedded in an extracellular polymeric matrix [4]. Persisters are highly-specialised cells that can be part of the biofilm and are able to survive an antibacterial treatment that results in killing of most of the bacterial population [5]. Despite their importance in treatment failure, the actual mechanisms involved in the formation and maintenance of these persister cells in biofilms are still largely unknown.

*B. cenocepacia* strain J2315 has a large multi-replicon genome (8.06 Mb) and the function of a large fraction of genes annotated as “hypothetical” or “conserved hypothetical” is still unknown [6]. While previous research has predominantly focussed on larger proteins, evidence is accumulating that genes encoding polypeptides with a length between 10 and 200 amino acids (but usually smaller than 100 amino acids) are ubiquitous in the genomes of all living organisms and are involved in various biological processes [7,8]. These small proteins are found in nearly all subclasses of functional groups of the COG database and on average approximately 10% of the protein-coding genes belong to this group [9]. Some well-known small proteins include chaperonin Hsp 10, translation initiation factor IF-1 and several ribosomal proteins (e.g. S17, S19, L27 and L30). Despite their potential involvement in important cellular processes, the exact function of the majority of small proteins is still unknown.

Toxin antitoxin (TA)-modules form a special group of small proteins known to be involved in various essential cellular processes including cell cycle control and biofilm formation [10]. Type II TA-modules generally encode small proteins and consist of a toxin, which can inhibit an important cellular function and an antitoxin which can form a complex with the toxin, thereby inactivating it [11]. Those modules are negatively auto-regulated by binding of the antitoxin alone or the toxin-antitoxin combination to the promoter sequence. Toxins are typically between 31 and 204 amino acids long whereas antitoxins consist of 41 up to 206 amino acids [12].

The goal of the present study is to elucidate the role of selected small proteins in *B. cenocepacia* biofilm formation, persistence and virulence.

## 2 Material and methods

### 2.1 Strains and culture conditions

The strains and plasmids used in the present study are shown in Table 1. Strains were cultured at 37 °C on Luria-Bertani agar (LBA, Oxoid). Overnight cultures were diluted in Luria-Bertani broth (LBB, Oxoid) and incubated aerobically at 37 °C. Where appropriate, the following antibiotics were added for plasmid selection: chloramphenicol (Cm) (Sigma-Aldrich), gentamicin (Sigma-Aldrich), kanamycin (Sigma-Aldrich) and trimethoprim (Tp) (Ludeco). Overexpression mutants were grown in LBB supplemented with Tp at 800 μg/ml with or without 0.2% (w/v) rhamnose (Sigma-Aldrich).

**Table 1:** Strains and plasmids used in the present study. Tp^R^: trimethoprim resistance marker, Cm^R^: chloramphenicol resistance marker.

Strain	Information	Source (reference)
*B. cenocepacia*		
J2315 (LMG16656)	CF patient, UK	BCCM/LMG Bacteria Collection
Overexpression mutants		
Vector control	J2315 pScrhaB2 empty vector, Tp^R^	[27]
	J2315 pScrhaB2 BCAL0008a, Tp^R^	This study
	J2315 pScrhaB2 BCAL0683, Tp^R^	This study
	J2315 pScrhaB2 BCAL2532, Tp^R^	This study
	J2315 pScrhaB2 BCAL2734, Tp^R^	This study
	J2315 pScrhaB2 BCAL3186, Tp^R^	This study
	J2315 pScrhaB2 BCAM0271, Tp^R^	This study
	J2315 pScrhaB2 BCAM0971, Tp^R^	This study
	J2315 pScrhaB2 BCAM2623, Tp^R^	This study
	J2315 pScrhaB2 pBCA050, Tp^R^	This study
eGFP translational fusion reporters		
Vector control	J2315 pJH2 empty vector, Cm^R^	[24]
	J2315 pJH2 BCAL0008a, Cm^R^	This study
	J2315 pJH2 BCAL0683, Cm^R^	This study
	J2315 pJH2 BCAL2523, Cm^R^	This study
	J2315 pJH2 BCAL2734, Cm^R^	This study

	J2315 pJH2 BCAM0271, Cm^R^	This study

	J2315 pJH2 BCAM2623, Cm^R^	This study

*E. coli* DH5α		BCCM/LMG Bacteria Collection
*E. coli* DH5α BCAM0271-2	pScrhaB2 BCAM0271-2, Tp^R^	This study
*E. coli* helper	pRK2013	[26]
*E. coli* DJ624Δara BCAM0272	pBAD33 BCAM0272	This study
*E.coli* DJ624Δara	MG1655, lac Iq, Δara	[44]
**Plasmids**		
pBAD33	P15A, Cm^R^, pBAD promoter	[44]
pJH2	promoter *eGFP* replaced with multiple cloning site, Cm^R^	[24]
pScrhaB2	*ori*pBBR1*rhaR*, *rhaS*, *PrhaB* Tp^R^*mob*+	[27]

### 2.2 Identification of small proteins with unknown function

The *Burkholderia* genome database was used to select proteins of 20–100 amino acids in size that were annotated as hypothetical [13]. Using BLASTP we searched for similarity with non-hypothetical proteins in other bacteria and conservation in *Burkholderia*. The cut-off *E*-value and the identity threshold used in this analysis were 10^−5^ and 40%, respectively. Only well annotated genomes were used for assessing conservation [14]. These included for the *Bcc*: *Burkholderia cenocepacia* K56-2, H111, AU1054, HI2424 and MC0-3, *Burkholderia lata* 383, *Burkholderia vietnamiensis* G4, *Burkholderia multivorans* ATCC 17616, *Burkholderia ambifaria* AMMD, *Burkholderia contaminans* MS14, *Burkholderia dolosa* AU0158, *Burkholderia cepacia* GG4 and *Burkholderia pyrrocinia* DSM 10685; for the *Burkholderia pseudomallei* group: *Burkholderia thailandensis* E264, *Burkholderia pseudomallei* K96243, *Burkholderia mallei* ATCC 23344; and for the other *Burkholderia* species: *Burkholderia xenovorans* LB400, *Burkholderia phytofirmans* PsJN, *Burkholderia gladioli* BSR3, *Burkholderia phymatum* STM815, *Burkholderia glumae* BGR1 and *Burkholderia rhizoxinia* HKI 454. Proteins were considered to be conserved if present in at least 11 of the 13 searched *Bcc* species, in all species of the *B. pseudomallei* group or in 5 of the 6 species of the other *Burkholderia* species [14].

### 2.3 Expression of small proteins

Available transcriptomic datasets [3,[15], [16], [17], [18]] were used to determine which small proteins are expressed during various stress conditions. Based on Van Acker et al. (2014) small proteins highly expressed in biofilms were selected [17]. In this study the number of reads assigned to a transcript was divided by the transcript length and normalized to the number of mapped reads to obtain reads per kb per million (RPKM) expression values. Genes with an RPKM >200 were considered highly expressed. For these proteins the transcription start site was determined based on published data from differential RNA sequencing [19]. To confirm expression of these genes, two different approaches were used, LC-MS based proteomics and use of translational fusion reporters in which the gene encoding a putative small protein was fused with an eGFP-encoding gene.

### 2.4 LC-MS based proteomics

Biofilms were grown as described below. After 24 ​h cells were harvested by vortexing and sonication (2 ​× ​5 ​min) (Branson 3510, Branson Ultrasonics Corp, Danbury, CT) and transferred to falcon tubes. Falcon tubes were subsequently centrifuged for 9 ​min ​at 5000 ​rpm and the supernatant was removed. Protein extraction was performed by using the R1-R3 extraction kit (Bio-Rad). For this, the cell pellet was resuspended in 500 ​μL of R1 buffer supplemented with benzonase (1 ​μl/ml) and protease inhibitor cocktail (1 x), and 0.5 ​mg/ml lysozyme, 1 ​mM dithiothreitol (DTT). The samples were sonicated for 10 ​min followed by centrifugation (5 ​min, 15 ​000 ​rpm). The supernatant was removed and vacuum-dried in a vaccuum concentrator (CentriVap, Labconco). The remaining pellet was dissolved in ready-prep R3 buffer (Biognosys). After vortexing and centrifuging the sample, the supernatant was removed and vacuum-dried. In order to enrich the small proteins in the samples, SDS-PAGE was performed on a Criterion TGX 4–15% gel (Bio-Rad). The samples were resuspended in 10 ​μl Laemmli buffer (R1+R3 pooled) and 1 ​μl β-mercaptoethanol followed by incubation for 10 ​min at 95 ​°C. The denaturated samples were loaded on the gel together with a Precision Plus Protein All Blue Standard (Bio-Rad). After running the gel for 30 ​min at 150 ​V and 60 ​min at 200 ​V, a cut-off point for the small proteins was chosen at 25 ​kDa. To obtain peptides suitable for LC-MS-analysis, the small proteins were digested in-gel as described before [20]. Briefly, the gel-pieces were washed three times for 10 min with a 25 mM tri-ethyl ammoniumbicarbonate (TEABC), 50% (v/v) acetonitrile (ACN) solution, followed by reduction of the proteins with 10 mM DTT in 25 mM TEABC for 1 h at 56 °C and alkylation with 200 mM S-methyl-methanethiosulfonate (MMTS) for 1 h at room temperature. After performing another wash-step, the gel-pieces were dehydrated with 100% ACN (2x) and vacuum-dried. Next, tryptic digest was performed by incubating the gel-pieces overnight at 37 °C in a 10 ng/μl tryptic solution (1 mM CaCl_2_, 5% (v/v) ACN and 50 mM TEABC). The supernatant was removed and vacuum-dried. The remaining gel-pieces were subjected to a sequential peptide extraction with an increasing amount of ACN (50%-75%–100% (v/v)) in 25 mM TEABC for 30 min, after which, each supernatant was pooled with the first (vacuum-dried) supernatant. The peptide samples were subsequently dissolved in 20 μl 0.1% formic acid and spiked with Hi3 *E. coli* standard (Waters) to a final concentration of 25 fmol/μl. Equal fractions of all samples were pooled to generate quality control samples to assess system stability during the LC-MS analysis and to enhance alignment during data-analysis. The LC separation was performed by a nanoACQUITY UPLC system (Waters) equipped with a trap column (180 μm × 20 mm nanoACQUITY UPLC 2G-V/MTrap 5 μm Symmetry C18, Waters) and an analytical column (100 μm × 100 mm nanoACQUITY UPLC 1.7 μm Peptide BEH, Waters). The system was operated with running buffer A (0.1% formic acid, 3% DMSO) and B (0.1% formic acid, 99.9% ACN) to enable gradient elution of the peptides according to their physicochemical properties. Trapping mode was used, which implies that peptides were first collected in the trap column at a flow rate of 8 μl/min for 2 min with 99.5% buffer A (full retention), followed by transfer to and separation on the analytical column with a 60 min gradient elution profile from 3% to 40% buffer B, at a flow rate of 0.3 μl/min. MS data acquisition was performed by an ESI-Q-TOF Synapt G2-Si (Waters), operated in positive mode. To ensure maximum coverage of the small proteins, all samples were analysed label-free with both data-dependent and data-independent acquisition (DDA and DIA). For DDA, full scan MS and MS/MS spectra (*m*/*z* 50-5000) were acquired in sensitivity mode. The survey MS scans were acquired using fixed scan times of 200 ​ms. The subsequent tandem mass spectra were acquired on fragment ions with a minimum intensity of 2 000 cps, derived from maximum eight precursors with a charge state 2 ​+ ​or higher, using collision induced dissociation. MS/MS scan time was set to 100 ​ms with an accumulated ion count of 200.000 cps. Dynamic exclusion of the fragmented precursor ions was set to 30 ​s. Ion mobility wave velocity was ramped from 2500 to 400 ​m/s in MS/MS to enable wideband enhancement in order to obtain a near 100% duty cycle on singly-charged fragment ions, so called HD-DDA. For DIA, both low- and high-energy (i.e. precursor- and fragment ions) scans (*m*/*z* 50-2000) were acquired alternately using fixed scan times of 600 ms, so-called ultradefinition MS^E^ acquisition [21]; for the high-energy scans, an in-house optimised collision energy look-up table was used. Ion mobility wave velocity was ramped from 1200 to 350 m/s. For both acquisitions, a simultaneous lock spray on glufibrinopeptide-B (*m*/*z* 785.8427) was acquired at a scan rate of 30 s to enable m/z-calibration.

Both DDA and DIA datasets were imported into Progenesis QIP (Non-linear Dynamics). After *m*/*z* calibration, peak picking, and alignment of the different samples, an *.mgf file was exported from the DDA-analysis and imported into the Mascot Daemon search engine. To identify the proteins, a database search was performed against *Burkholderia* [13] supplemented with contaminants from the cRAP Database [22] and the small proteins. For this, parameters were set to a peptide mass tolerance of 10 ​ppm, fragment mass tolerance of 0.3 ​Da, and trypsin as enzyme specificity; methylthio on cysteine was set as fixed modification and deamidation of asparagine/glutamine and oxidation of methionine were set as variable modifications. A SynaptG2Si instrument was defined in-house to only account for singly-charged ions as these are the only ones generated during HDDDA acquisition. Subsequently, the identifications were exported as an *.xml file and imported back into Progenesis QIP to match the identifications with the peptide ion intensities. The DIA-data was identified using the Ion Accounting (IAdb) search algorithm imbedded in Progenesis QIP. The mass tolerance was set to automatic, enzyme specificity and modifications were kept identical to the previous search, as well as the supplemented *Burkholderia* database. The mass spectrometry proteomics data have been deposited to the ProteomeXchange Consortium via the PRIDE [23] partner repository with the dataset identifier PXD011198 and 10.6019/PXD011198.

### 2.5 Construction of eGFP translational fusion reporters in *B. cenocepacia*

Translational GFP reporter fusion plasmids [24] were constructed and the production of an eGFP (“enhanced” GFP) protein was used as a marker of small protein expression. Six genes with identified transcription start sites (TSS) were chosen from our list of genes that are highly expressed in biofilms (BCAL0008a, BCAL0683, BCAL2532, BCAL2734, BCAM0271 and BCAM2623). The 5′UTR plus approx. 150 nucleotides, presumably containing the native promoter region, upstream of the TSS and up to 50 nucleotides of the coding region were amplified by PCR using a Phusion High Fidelity PCR Kit (Bioké NEB). The primers and annealing temperature are listed in Table S1. Cycling conditions were 30 s at 98 °C, 30 cycles of 10 s at 98 °C, 30 s at 60 or 65 °C, 24 s at 72 °C, and finally 10 min at 72 °C. PCR products were purified with a Nucleospin Gel and PCR Clean-up (Macherey-Nagel), digested with NdeI (Promega) and BglII (Promega), purified, and subsequently ligated into a plasmid pJH2 [24], containing a Cm selection marker and the eGFP sequence lacking the startcodon. The CaCl_2_ method was used to transform the plasmid into *E. coli* DH5α [25]. Resistant colonies were isolated and screened for the presence of the construct. Plasmids were transferred into *B. cenocepacia* J2315 by triparental mating using pRK2013 as a helper plasmid [26]. Exconjugants were selected on LBA plates supplemented with 200 μg/ml Cm and 50 μg/ml gentamicin and screened. Plasmid extraction, PCR (primers in Table S1) and agarose gel electrophoresis were performed to determine the presence of the correct insert. In addition, Sanger sequencing was performed for the reporters for which no fluorescence could be observed.

### 2.6 Flow cytometry

To determine the expression of small proteins by *B. cenocepacia*, the eGFP production from small protein translational fusion reporters was determined using flow cytometry analysis. To this end, biofilms of the different eGFP translational fusion reporters were grown as described below. After 24 h cells were washed to remove non-adherent cells, harvested by thoroughly pipeting up and down and transferred to a new 96 well microtiter plate. Cell supensions were diluted 1/1000 to obtain approx. 10^6^ CFU/ml and analysed by flow cytometry (Attune NxT, Life Technologies). Bacteria were defined based on the forward and side scatter signal, and a threshold was set to exclude non-cellular particles and cell debris. Excitation wavelength was 488 nm and fluorescence emission was detected through a 530/30 bandpass filter. At least 10,000 bacteria were analysed per sample and the average eGFP signal in the bacterial population was determined. Two wells were included per condition and the experiment was repeated twice (n = 3 × 2).

### 2.7 Construction of *B. cenocepacia* overexpression mutants

To study the role of different small proteins, overexpression mutants were constructed in *B. cenocepacia* J2315 (LMG16656) as described previously [3]. The primers and specific cycling conditions are listed in Table S1. Cycling conditions were 30 s at 98 °C, 30 cycles of 10 s at 98 °C, 30 s at 55 °C, 60 °C or 65 °C, 24 s at 72 °C and finally 10 min at 72 °C. PCR-products were digested using NdeI and XbaI and ligated into a plasmid pSCrhaB2 [27], containing a rhamnose-inducible promotor and a Tp selection marker.

### 2.8 Measurement of planktonic growth

To study the influence of small proteins on growth, overnight cultures (16 h, 250 rpm) of the constructed overexpression mutants were diluted to an optical density (λ = 590 nm) of 0.05 (approx. 5 × 10^7^ CFU/ml) in LBB supplemented with 0.2% (w/v) rhamnose. 50 μl of this suspension was added to the wells of a 24 well microtiter plate (TPP) and mixed with 950 μl LBB supplemented with 0.2% (w/v) rhamnose. To study growth in different conditions, 10 μl of the suspension was added to the wells of a 96 well U-shaped microtiter plate (TPP) and mixed with 190 μl medium. Eight different media were tested: LBB, 1/10 diluted LBB, LBB set to pH 4.2 or 8.2, and LBB with 1.5% (w/v) NaCl, 0.015% (w/v) SDS, 0.045% (w/v) NaOCl, or 0.25 mM 2,2′-bipyridyl. Plates were incubated at 37 °C and the absorbance was measured at 590 nm every 30 min for three days in an Envision multilabel plate reader (PerkinElmer). One to three wells per strain were included in the experiments with a 24- or 96-well microtiter plate, respectively, and the experiment was performed twice.

### 2.9 Biofilm formation

To measure differences in biomass between vector control and overexpression mutants, an inoculum suspension containing approx. 5 × 10^7^ CFU/ml was added to the wells of a round-bottomed 96-well microtiter plate (TPP). Ten wells per strain were included and the experiment was performed at least three times. Biofilms were grown in LBB supplemented with Tp and 0.2% (w/v) rhamnose to induce expression of the respective small proteins. Following four hours of adhesion, the supernatant was removed and the plates were rinsed with physiological saline (0.9% w/v NaCl,PS). Subsequently, 100 μl of fresh LBB supplemented with Tp and 0.2% (w/v) rhamnose was added and the plates were further incubated at 37 °C. After 24 h, the supernatant was removed, wells were rinsed with 100 μl PS and 100 μl of a 99% (v/v) methanol solution (Sigma) was added for 15 min. Methanol was removed and plates were dried at 37 °C. When all residual methanol was evaporated, 100 μl of a 0.1% (v/v) crystal violet stain (Prolab Diagnostics) was added for 20 min. Plates were rinsed with water and 150 μl of a 33% acetic acid solution was added for five minutes. After shaking, absorption was measured at 590 nm [28].

### 2.10 Confocal laser scanning microscopy

Biofilms of the vector control and overexpression mutants were grown in wells of a black 96-well plate with glass bottom (Greiner Bio-one) in LBB supplemented with Tp and rhamnose as described above. After 24 h of growth, the wells were rinsed with PS and filled with 100 μl staining solution (containing 1 ml PS, 3 μl SYTO9 and 3 μl propidium iodide). The plates were incubated in the dark for 15 min at room temperature and the biofilm was visualized with a Nikon C1 confocal laser scanning module attached to a motorized Nikon TE2000-E inverted microscope (Nikon Benelux) equipped with a CFI Plan Apo VC 60 × 1.4 NA oil immersion objective (Nikon) to obtain fluorescent images and Z-stacks. A 488 nm continuous wave laser (Coherent Sapphire) was used for excitation of SYTO9. Images were obtained from at least two biofilms for each strain and representative images are shown.

### 2.11 Determination of the minimal inhibitory concentration (MIC)

MICs were determined in duplicate according to the EUCAST broth microdilution protocol using flat-bottom 96-well microtiter plates (TPP) [29]. Tobramycin (Tob) (TCI Europe), meropenem (Mer) (Astrazeneca) and ciprofloxacin (Cip) (Sigma Aldrich) concentrations tested ranged from 0.25 to 1024 μg/ml (Tob) and from 0.25 to 128 μg/ml (Mer, Cip). The MIC was defined as the lowest concentration for which no significant difference in optical density (λ = 590 nm) was observed between the inoculated and blank wells after 24 h incubation. All MIC determinations were performed in duplicate.

### 2.12 Quantification of persister cells

To determine the number of surviving persisters, 24 h old biofilms were exposed to Tob or Cip in a concentration of 4 x MIC (1024 or 32 μg/ml, respectively) for 24 h [3]. Biofilms were grown as described above. After 24 h of growth, 120 μl of an antibiotic solution in PS or 120 μl PS (control) was added and the plates were incubated for an additional 24 h at 37 °C. Twelve wells were included per condition. Cells were harvested by vortexing and sonication (2 × 5 min) (Branson 3510, Branson Ultrasonics Corp) and quantified by plating on LBA (n ≥ 3 for all experiments).

### 2.13 Adhesion, invasion and intracellular growth/survival in lung epithelial cells

A549 lung epithelial cells (ATCC CCL-185) were maintained in GTSF-2 medium supplemented with 2.5 mg/l insulin transferrin selenite (ITS, Sigma-Aldrich), 1.5 g/l sodium bicarbonate, and 10% (v/v) heat inactivated foetal bovine serum (FBS, Life Technologies) at 37 °C under 5% CO_2_. 2.5 × 10^4^ A549 cells were added per well in 24-well plates (1 ml volume) (SPL Life Sciences), and incubated until confluency was reached (96 h). At the time of infection, the monolayer was rinsed three times with Hanks’ Balanced Salt Solution (HBSS, Life Technologies) and cell culture medium without FBS was added. For studies involving the induction of small proteins, 0.2% (w/v) rhamnose was added to the cell culture medium. Overnight cultures of the vector control or the different small protein overexpression mutants were resuspended in cell culture medium and added to the cells at a multiplicity of infection (MOI) of approx. 100:1. To investigate the influence of the different small proteins on adhesion, invasion and intracellular survival, an antibiotic protection assay was developed. After 2 h of infection, the wells were rinsed twice with HBSS followed by either 1) the addition of 1% (v/v) Triton X-100 to lyse host cells, vigorous mixing and plating to determine the number of adhered and/or invaded cells or 2) the addition of an antibiotic solution containing 1 mg/ml amikacin, ceftazidime and meropenem in cell culture medium for 2 h to kill the extracellular bacteria. After treatment, cells were again rinsed twice with HBSS followed by 1) the addition of 1% (v/v) Triton X-100, vigorous mixing and plating to determine the number of invading bacteria or 2) the addition of an antibiotic solution containing 0.01 mg/ml amikacin, ceftazidime and meropenem to inhibit growth of extracellular bacteria. After 24 h, the remaining wells were washed, followed by the addition of 1% (v/v) Triton X-100, vigorous mixing and plating to determine intracellular growth/survival. As a control to ensure the absence of extracellular bacteria, the supernatant was plated. Plates were incubated for 48 h to determine the CFU/ml (n = 4).

To determine the fraction of adhering cells, the number of CFU recovered two hours after infection was compared to the initial inoculum. The fraction of cells capable of invading epithelial cells was determined by comparing the number of adhering cells (determined 2 h after infection) with the number of cells recovered after an additional 2 h treatment with antibiotics (= 4 h post infection). The number of cells capable of prolonged intracellular survival (or even intracellular growth) is calculated based on the number of CFU recovered after 24 h compared to the number of CFU recovered 4 h after infection.

### 2.14 Fluorescence microscopy of infected A549 lung epithelial cells

Expression of the small proteins by *B. cenocepacia* in the presence of A549 cells was evaluated after 24 h infection using the translational fusion reporters. To this end, A549 cells were infected and treated as described above for the antibiotic protection assay, to determine the expression of small proteins in the intracellular bacteria (24 h time point). After 24 h infection, the cells were washed and fluorescent and light microscopic images were taken using an EVOS FL Auto Imaging System (Life Technologies) at 300 x magnification. The same settings were used to record pictures of cells infected with the different reporter strains. Experiments were performed in biological and technical duplicates and a representative image is shown.

### 2.15 Cytotoxicity assay

To investigate the influence of the different small protein overexpression mutants on cytotoxicity, cell viability was evaluated based on a lactate dehydrogenase (LDH) assay. The LDH activity assay kit (Sigma) was used to measure the release of cytosolic LDH by the lung epithelial cells following exposure to vector control and overexpression mutants. Medium from monolayers infected at an MOI of 100 for 48 h was centrifuged for 15 min at 3700 rpm. The supernatant was used for LDH quantification following the manufacturer's instructions. A standard curve using NADH was included. As a positive control, lung epithelial cells were lysed with 1 ml 1% (v/v) Triton-X100. The experiments were performed in triplicate (n = 3 × 2). The data are presented as a percentage of LDH release from the positive control.

### 2.16 Effect of expression of BCAM0271-BCAM0272 in *E. coli* on growth

To investigate whether BCAM0271-2 encodes a TA-module, plasmids containing either the toxin BCAM0272 alone or both genes were constructed, transformed into *E. coli* DJ624Δara or DH5α, respectively, after which growth was evaluated. The primers are listed in Table S1. Cycling conditions were 120 s at 98 °C, 30 cycles of 10 s at 98 °C, 20 s at 62 °C, 30 s at 72 °C for BCAM0272 and 30 s at 98 °C, 30 cycles of 10 s at 98 °C, 30 s at 55 °C, 24 s at 72 °C and finally 10 min at 72 °C for BCAM0271-2. PCR-products were digested using XbaI and PstI (BCAM0272) or NdeI and XbaI (BCAM0271-2) and ligated into plasmid pBAD33 with Cm selection marker and downstream of a pAra promoter (BCAM0272) or into plasmid pSCrhaB2 [27], containing a rhamnose-inducible promotor and a Tp selection marker (BCAM0271-2). *E. coli* DJ624Δara pBAD33 BCAM0272 was grown in the presence of 1% (w/v) glucose to ensure repression of BCAM0272. To study the influence on growth when BCAM0272 was expressed alone or in combination with BCAM0271, overnight cultures of the different mutants were diluted to an optical density (λ = 590 nm) of 0.05 (approx. 5 × 10^7^ CFU/ml) in LBB supplemented with Cm (BCAM0272) or Tp (BCAM0271-2). This culture was further diluted up to 10^−8^ and 10 μl of each dilution was streaked on LBA supplemented with the appropriate antibiotics with or without 0.2% (w/v) arabinose (BCAM0272) or rhamnose (BCAM0271-2). Plates were incubated at 37 °C.

Assessing tRNA acetylation and synthesis of GFP-StrepII reporter protein expressed from the T7 promoter in an *in vitro* transcription-translation system.

The DNA fragment for synthesizing the BCAM0272 toxin *in vitro* in a coupled transcription-translation reaction (PurEXPRESS, NEB) was amplified using oligos 5′UTR-BCAM0272 (GCGAATTAATACGACTCACTATAGGGCTTAAGTATAAGGAGGAAAAAATATGAGCGGTGCGCAGTTGG) and 3′UTR-BCAM0272-strepII (AAACCCCTCCGTTTAGAGAGGGGTTATGCTAGTTATTATTTTTCGAACTGCGGGTGGCTCCACTTCACCGTTGCCAATGGCAT). The amplification conditions were 120 s at 98 °C, 30 cycles of 10 s at 98 °C, 20 s at 65 °C, 30 s at 72 °C. The fragment was then purified on a PCR purification column. 10 μl of coupled *in vitro* transcription-translation reaction was supplemented with 100 ng of DNA fragment coding T7-BCAM0272 toxin. After 1 h of synthesis reaction at 37 °C in one of the reactions 0.1 mM of [^14^C]-acCoA was added and synthesis and acetylation were allowed to proceed for additional 30 min. 2 μl of reaction was subjected to 10% TBE-polyacrylamide gel electrophoresis for 40 min at 120 V. Gel was then stained with 0.2% methylene blue solution and destained with water. The gel was dried and exposed to a phosphor imaging screen overnight and visualised with Amersham Typhoon Phosphor imager (GE).

DNA coding for GFP-strepII was amplified with 5′UTR-GFP (GCGAATTAATACGACTCACTATAGGGCTTAAGTATAAGGAGGAAAAAATATGAGTAAAGGAGAAGAACTTTTCAC) and 3′UTR-GFP-strep (AAACCCCTCCGTTTAGAGAGGGGTTATGCTAGTTATTATTTTTCGAACTGCGGGTGGCTCCATTTGTATAGTTCATCCATGCCA) oligonucleotides. The amplification conditions were 120 s at 98 °C, 30 cycles of 10 s at 98 °C, 20 s at 65 °C, 30 s at 72 °C. The BCAM0272-strepII toxin was produced in 100 μl of *in vitro* transcription-translation reaction and purified from *in vitro* translation reaction using streptactin-agarose beads. *In vitro* translation reactions for synthesis of reporter protein were set up using 100 ng of template DNA, 0.1 μM of toxin, and one of the reactions was supplied with 0.1 mM acCoA. Reactions were incubated for 2 h at 37 °C and 5 μl of reactions were resolved by SDS-PAGE gel electrophoresis followed by western blot with anti-strepII antibodies.

### 2.17 Statistical data analysis

Statistical data analysis was performed using SPSS software, version 21 (SPSS). The Shapiro-Wilk test was used to verify the normal distribution of the data. Normally distributed data were analysed using a one-sample *t*-test or an independent sample *t*-test, while non-normally distributed data were analysed using a Wilcoxon signed-rank test or a Mann-Whitney test. P-values < 0.05 were considered significant.

## 3 Results and discussion

### 3.1 Identification of small proteins

Small proteins are defined as polypeptides with a length of 10–200 amino acids, but are usually smaller than 100 amino acids. The average proportion of genes encoding proteins smaller than 200 amino acids among all annotated bacterial and archaeal genes is approx. 10% [9]. In the *B. cenocepacia* J2315 genome we found that 2084 of the 7115 protein-coding genes (29.3%) were smaller than 600 base pairs, which is close to the so-far highest described fraction of small proteins (33.4% in the alphaproteobacterium *Anaplasma phagocytophilum*) [9]. 8.0% of the *B. cenocepacia* J2315 protein coding genes (572) were smaller than 300 base pairs. These genes smaller than 300 base pairs belong to very diverse functional categories (Table S2), and 345 of these genes are currently annotated as hypothetical. For 234 of them no similarity could be found with known genes in other bacteria using BLAST (criteria: E < 10^−5^ and identity > 40%). However, the majority of the genes encoding hypothetical proteins are differentially expressed in various conditions [3,15,16], suggesting these proteins have a specific physiological function (Table S2).

Based on available RNA sequencing data [17] a list of 27 small proteins highly expressed in *B. cenocepacia* J2315 biofilms (i.e. RPKM value > 200) was compiled for further analysis (Table 2). While no similarity could be found with non-hypothetical genes in other bacteria and only five are annotated as ‘conserved hypothetical’, homologs of 20 of them are present in other *Bcc* species. For four of these, homologs are also found in species belonging to the pseudomallei group (i.e. *B. pseudomallei, B. thailandensis* and *B. mallei*) and in other *Burkholderia.* Four genes were identified only in the genomes of members of the pseudomallei group, while eight genes were only present in the genomes of members of the group of ‘other’ *Burkholderia* species (Table 2).

**Table 2:** Selected genes encoding small proteins highly expressed in biofilms. Tob: tobramycin, tbH_2_O_2_: *tert*-butyl peroxide CHX: chlorhexidine, BF: biofilm, PL: planktonic growth. Available transcriptomic data were used to determine which small proteins are expressed during various stress conditions [3,[15], [16], [17], [18]].

Gene	Length (bp)	Conserved in			Fold change compared to untreated cultures								Fold change BF vs. PL	RPKM in BF
		*Bcc*	*Bpm* group	Other *Burkholderia*	Tob	H_2_O_2_	tb H_2_O_2_	CHX	Low [Fe]	Low [O_2_]	Heat	pH = 4.2		
BCAL0008a^a^^,^^b^	230	+	+	+		−1.5			1.6					210
BCAL0193	233	+			10.1			6	1.6	1.6	2.7	2.7		442
BCAL0516	212	+								1.7		1.5		648
BCAL0683^a^^,^^b^	257	+		+	4.4	102.1	63.8			3.9	2.5	1.8		4135
BCAL1282	170	+	+		0.3	1.7	1.7			4.2	2			524
BCAL1747A	212	+				4.4	2.1		−1.9	3.1				458
BCAL2010	278	+				−1.5					1.6			203
BCAL2308	146	+		+		3.9				3		2.6		1547
BCAL2532^a^^,^^b^	212	+	+		0.4	1.6	1.7	0.5		−2.8	−1.7			433
BCAL2649	188	+					−1.6							329
BCAL2734^a^^,^^b^	224	+			3.7	3.7	1.8			14.9	2		33.6	491
BCAL3186^b^	230	+	+		0.4									713
BCAL3298	170						1.7			5.6				709
BCAM0271^a^^,^^b^	269				11.6	1.8				1.7				234
BCAM0895	176	+	+		5.4	14.2	1.6			28		5.7	3.2	427
BCAM0971^c^^,^^b^	269	+	+		0.2		−1.6			−2.1				619
BCAM1052^c^	176	+				1.6				1.6	1.5	1.7		490
BCAM1811^c^	224	+	+			−1.5				−1.9			−14.8	571
BCAM2207	278	+					2			2	6.5	1.9		608
BCAM2287	290	+								1.8	1.5	−1.5		227
BCAM2623^a^^,^^b^	263	+										−1.7		1198
BCAM2685	155	+				18.1	5		−1.5	21.9	−1.6	1.7		888
BCAS0244	206				0.2			0.2		−1.8	−2		−238.5	979
BCAS0245A	209	+											−248	2246
BCAS0535	224					−2.3				−2				213
BCAS0540b	236													339
pBCA050^b^	272						−2			−1.6			−3.4	201

a Genes for which eGFP translational fusion reporters were constructed.

b Genes for which overexpression mutants were constructed.

c Small proteins without an own transcription start site as determined with differential RNAseq [19].

None of these 27 proteins contain a signal peptide, suggesting they are not secreted. 22 small proteins have their own transcription start site as determined by differential RNAseq [17,19] (Table 2). BCAL2308 forms an operon with BCAL2309 encoding a putative copper related MerR family regulating protein. BCAM1811 seems to have its own transcription start site but is also in an operon with BCAM1810, encoding a putative cold shock protein. BCAM1052 forms an operon with BCAM1051, encoding a phage death-on-curing protein. BCAM0971 is part of a larger operon also containing genes encoding various subunits of succinate dehydrogenase (BCAM0966 – BCAM0970) and BCAM0972 (encoding a citrate synthase) and, was found to be essential in *B. cenocepacia* J2315 [30] and H111 [31].

### 3.2 Expression of small proteins

**Fig. 1:**
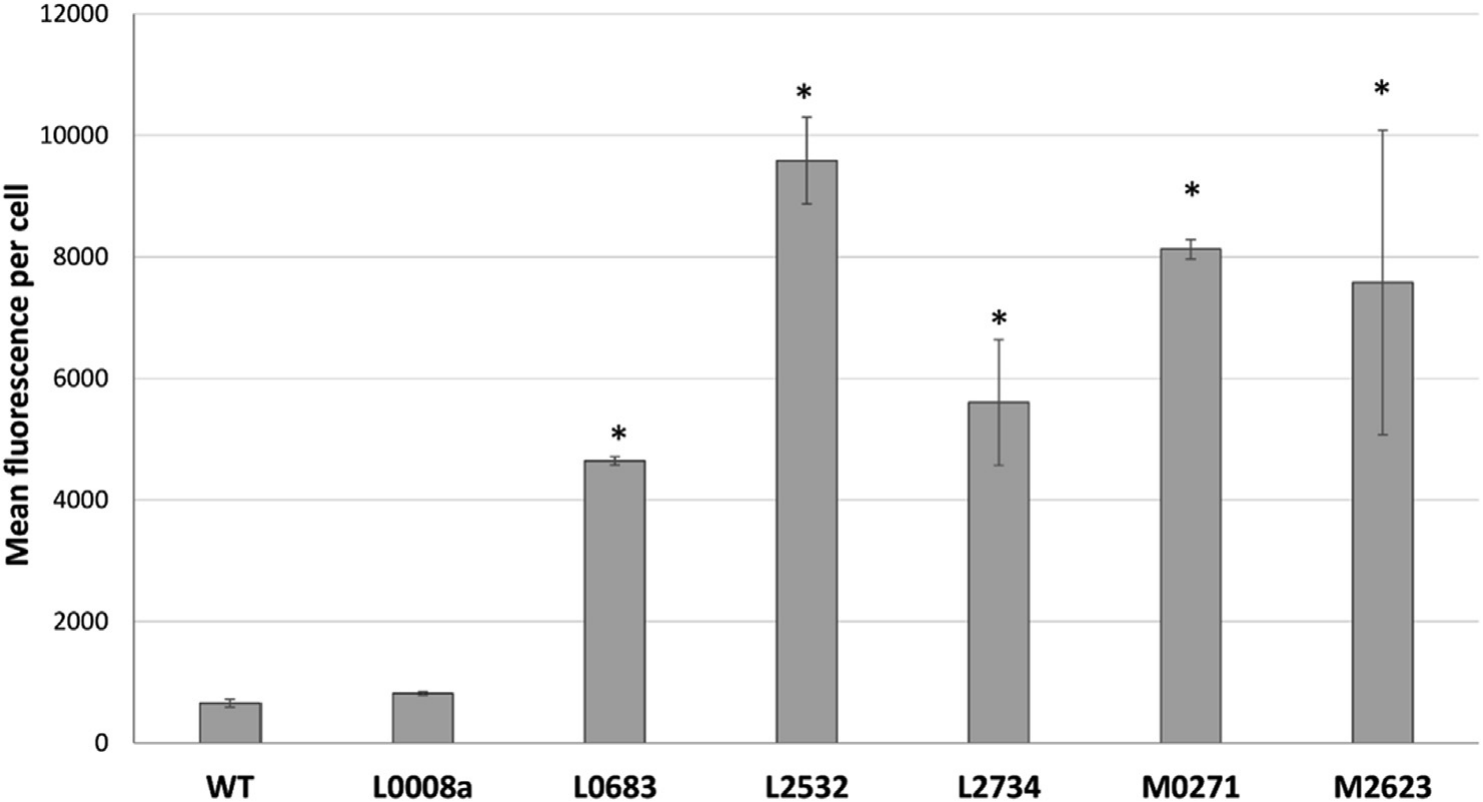
eGFP production from small protein translational fusions. eGFP derived fluorescence per cell (arbitrary fluorescence units) determined by flow cytometry for the different translational fusion reporters grown in a biofilm. Error bars represent SEM (n = 3 × 2). Statistically significant differences compared to WT (P < 0.05) are indicated by an asterisk.

Translation of small protein genes was assessed by LC-MS based proteomics and by constructing eGFP translational fusion reporters. Using LC-MS, expression of six of the 27 selected small proteins (BCAL0008a, BCAL3186, BCAM0271, BCAM0971, BCAM2623, pBCA050) could be confirmed in the DDA data (Table 3). In Table S3 a detailed overview of all 41 miniproteins identified by either the DDA or high-definition MS^E^ approach is given. Translational eGFP reporter fusion plasmids [24] were constructed for six small proteins with identified TSS. Five of these produced detectable eGFP in biofilms using flow cytometry analysis (Fig. 1). Only two of these five small proteins (BCAM0271 and BCAM2623) were also identified using LC-MS. On the other hand, BCAL0008a expression was confirmed by LC-MS, but no eGFP was produced from reporter fusions. To conclude, for nine small proteins translation could be confirmed by at least one approach. This suggests a biological role for these small proteins and highlights the importance of using different approaches to confirm production of small proteins.

**Table 3:** Genes confirmed as expressed by different approaches.

Gene	RNA sequencing	Proteomics	Translational fusion
BCAL0008a	Yes	Yes	No
BCAL0683	Yes	No	Yes
BCAL2532	Yes	No	Yes
BCAL2734	Yes	No	Yes
BCAL3186	Yes	Yes	Not tested
BCAM0271	Yes	No	Yes
BCAM0971	Yes	Yes	Not tested
BCAM2623	Yes	Yes	Yes
pBCA050	Yes	Yes	Not tested

To determine the role of the nine identified small proteins for which translation could be confirmed by at least one method (BCAL0008a, BCAL0683, BCAL2532, BCAL2734, BCAL3186, BCAM0271, BCAML0971, BCAM2623 and pBCA050), overexpression mutants, in which expression of the small protein is controlled by a rhamnose inducible promoter [27,32], were constructed and the effect of the overexpression on various phenotypes was determined.

### 3.3 Role of selected small proteins in growth and biofilm formation

**Fig. 2:**
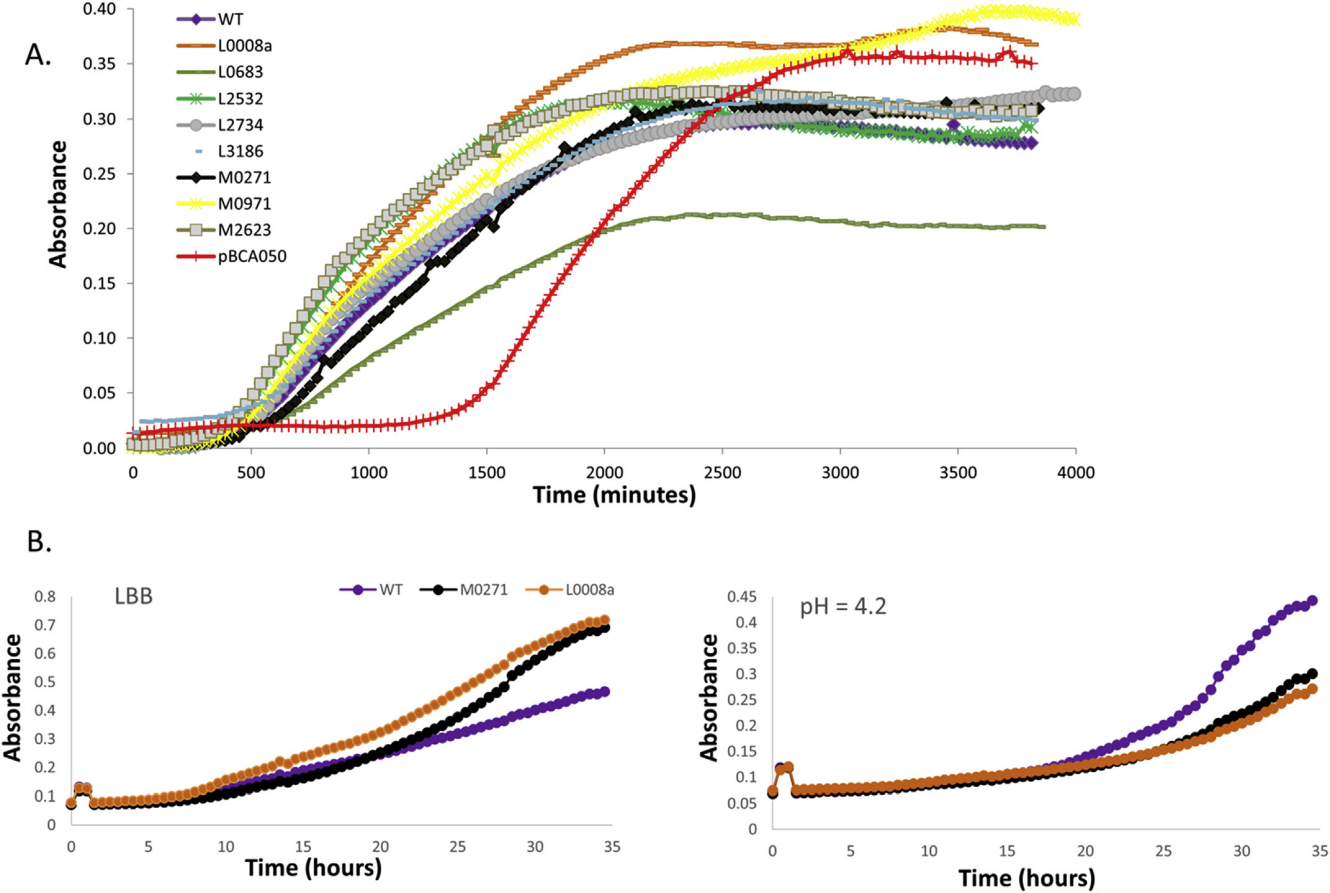
(a) Effect of overexpression of small proteins on the planktonic growth of *B. cenocepacia* J2315. Strains were grown in LBB supplemented with 0.2% (w/v) rhamnose. (b) Effect of overexpression of BCAM0271 and BCAL0008a on growth in LBB supplemented with 0.2% (w/v) rhamnose (left panel) or LBB pH 4.2 supplemented with 0.2% (w/v) rhamnose (right panel). Experiments were carried out twice and similar results were obtained. Curves from one experiment are shown. WT = vector control.

When grown planktonically in LBB, most overexpression mutants show a growth curve similar to WT (Fig. 2a). The pBCA050 overexpression mutant showed a prolonged lag phase, while the BCAL0683 overexpression mutant showed an increased doubling time (893 min vs. 550 min for WT) and grew to a lower maximal optical density in stationary phase. When overexpressed in *E. coli* DH5α, neither pBCA050 nor BCAL0683 had an effect on growth (data not shown), therefore, neither protein is generally toxic. Interestingly, for pBCA050 we observed antisense transcription overlapping the CDS [19], suggesting pBCA050 might be the toxin part of a type I TA-module. Type I TA-modules are composed of toxin protein and a small antisense RNA that plays the role of an antitoxin by controlling the expression of its toxin counterpart [33].

As many of the genes encoding for small proteins were differentially expressed in various growth conditions (Table 2), we investigated whether overexpression had an impact on growth under stress. Overall, stress affected WT and mutants similarly (Fig. S3), although minor differences occurred under specific stress conditions. For example, while the BCAL0008a and BCAM0271 overexpression mutants reached a slightly higher OD than WT after 32 h in LBB, they reached a lower OD than WT under acid stress (Fig. 2b).

**Fig. 3:**
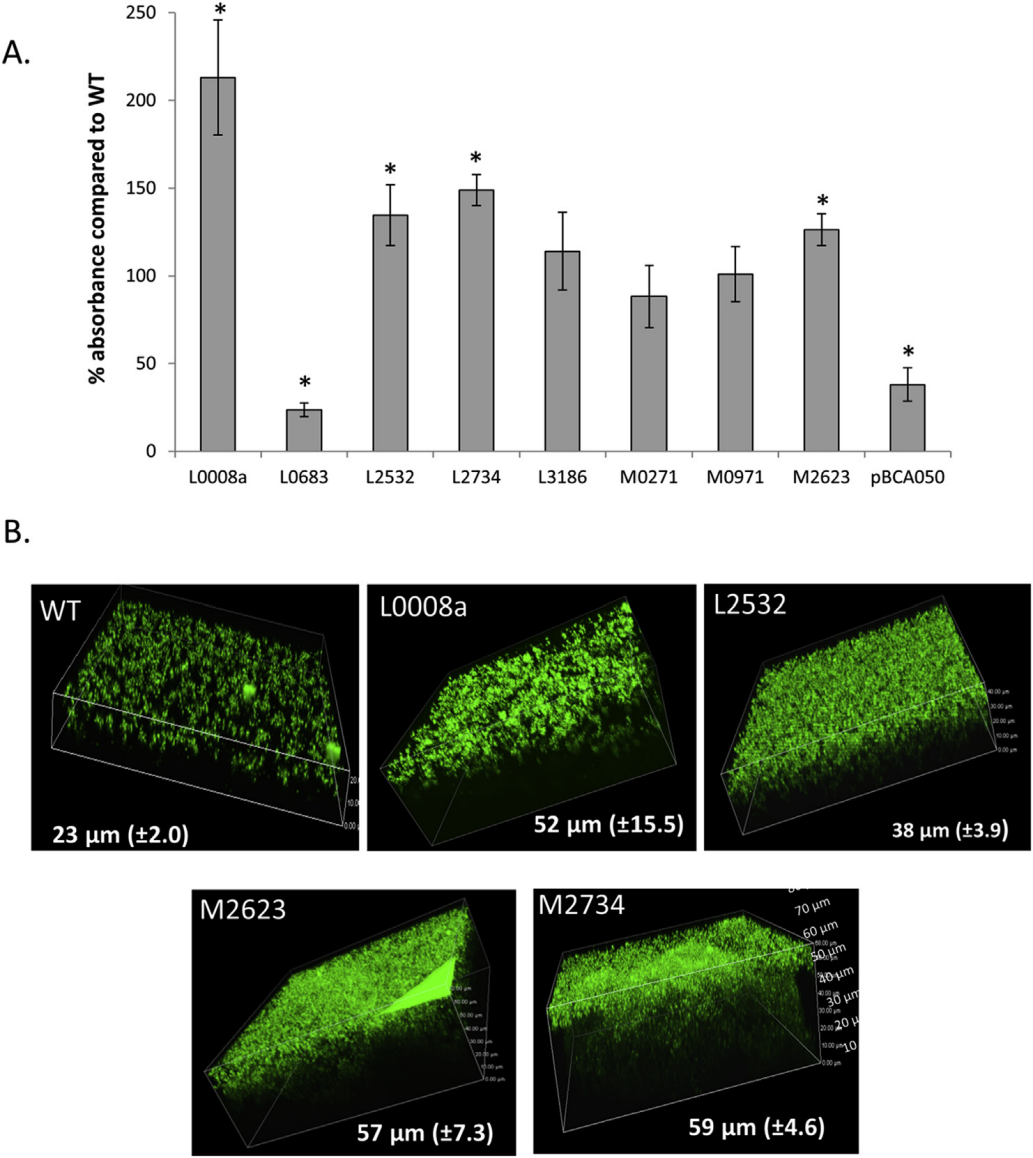
(a) Influence of small protein overexpression on biofilm formation using a crystal violet assay. The absorbance for WT (vector control) was 0.71 (SEM: 0.07). Error bars represent SEM (*n* ≥ 3). Statistically significant differences compared to WT (*P* < 0.05) are indicated with an asterisks. (b) Confocal laser scanning microscopy image of 24-h-old biofilms of *B. cenocepacia* J2315 vector control and the BCAM2623, BCAL2734, BCAL2532 and BCAL0008a overexpression mutants. The mean thickness of three experiments and SEM is indicated and a representative image is shown.

Biofilm biomass was quantified using crystal violet staining (Fig. 3a). A significant reduction was observed for the mutants overexpressing BCAL0683 or pBCA050 compared to the vector control, likely related to their growth phenotype. Overexpressing BCAL0008a, BCAL2532, BCAL2734 and BCAM2623 significantly increased biofilm biomass and this was confirmed using confocal scanning laser microscopy (Fig. 3b). Overall, the results show that these four small proteins play a role in biofilm development, while they have limited effect on planktonic growth.

### 3.4 Role of selected small proteins in antimicrobial susceptibility and persistence

**Fig. 4:**
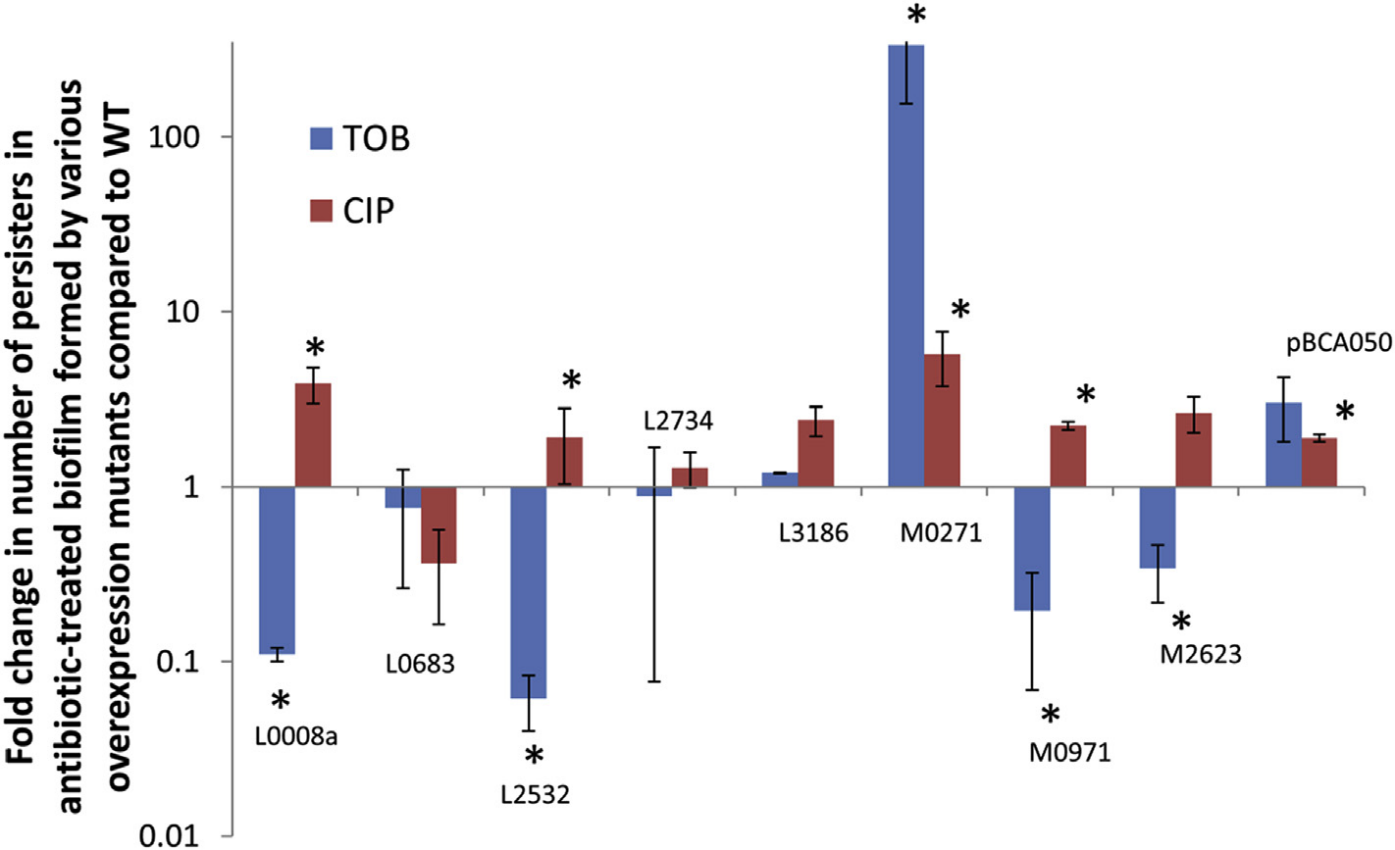
Number of surviving cells (persisters) in small protein overexpression mutants after treatment with high concentrations (4 x MIC) Tob or Cip. Data are expressed as the ratio of the fraction surviving persisters in the overexpression mutant and the fraction surviving persisters in the vector control. The fraction of surviving cells for the WT (vector control) was 0.03% (SEM: 0.02) and 4.16% (SEM: 1.26) after treatment with Tob or Cip, respectively. Error bars represent SEM (*n* ≥ 3). Statistically significant differences (*P* < 0.05) are indicated by an asterisk.

Overexpression of the nine small proteins did not change the MIC for tobramycin (MIC = 256 μg/ml), ciprofloxacin (MIC = 4 μg/ml) or meropenem (MIC = 8 μg/ml). However, overexpression of several small proteins affected the number of surviving cells recovered after treatment with high doses of tobramycin (persister cells) (Fig. 4). For three small proteins an interesting link between numbers of persisters in treated biofilms and expression patterns in biofilm was observed. BCAM0271 was upregulated after treatment with Tob [34] and a significant increase in persisters was observed after Tob treatment (335-fold increase) in the BCAM0271-overexpression mutant (Fig. 4). BCAM0971 and BCAL2532 were downregulated after treatment with Tob [34] and overexpression of these proteins significantly decreased the number of persisters after treatment with Tob, 5-fold and 16-fold decrease, respectively. We have previously shown that bacteria lower the production of NADH in the TCA cycle in order to reduce antibiotic-induced ROS production [3,35]. Whether BCAM0971 (part of a larger operon also containing genes encoding subunits of succinate dehydrogenase, an enzyme involved in the tricarboxylic acid cycle (TCA)), plays a role in metabolism remains to be determined, although its location, the downregulation upon Tob exposure and the decrease in cells surviving Tob exposure in the BCAM0971-overexpression mutant, suggest it does. For the BCAM0271 overexpression mutant, the number of persisters recovered after treatment of selected with high concentration of ciprofloxacin was also significantly increased (6-fold). For the other overexpression mutants differences in numbers of persisters after ciprofloxacin were small compared to the WT (Fig. 4) and it remains to be determined whether these small differences are biologically relevant. We had previously already shown that overexpression of BCAM0272 significantly increased persisters after treatment with Tob and Cip [34].

### 3.5 Optimization of the antibiotic protection assay to study intracellular *B. cenocepacia*

To evaluate the role of small proteins in the infection of human lung epithelial cells, the available antibiotic protection assay had to be modified. To quantify bacterial invasion in eukaryotic cells, a gentamicin protection assay is typically used but as *B. cenocepacia* is resistant to gentamicin, this method was not applicable. Martin and Mohr (2000) proposed a different approach in which cells are treated with a combination of 1 mg/ml ceftazidime and amikacin for 2 h, followed by an incubation period without antibiotics [36]. More recently Taylor et al. (2010) treated the cells for 3 h and added 1 mg/ml meropenem to the antibiotic mix [37]. We observed that when using a mix of the three antibiotics and 2 h of treatment, no surviving cells were detected (detection limit of five CFU/ml) in a planktonic culture with an initial inoculum of 5 × 10^7^ CFU/ml. However, when A549 epithelial cells infected with *B. cenocepacia* J2315 and treated with this combination were further incubated for an additional 22 h, we observed significant bacterial growth in the supernatant. This could be due to re-growth of surviving cells or due to growth of cells released by dead epithelial cells. To investigate this further, a rhamnose-inducible eGFP-producing mutant was used to infect the cells and a light and fluorescent microscopic image was taken. After 24 h bacterial cells and biofilm-like structures can be observed on the host cells, despite the initial 2 h exposure to antibiotics (Fig. S4). This indicates that extracellular bacteria survive the antibiotic treatment. To prevent extracellular growth, this experiment was repeated in the presence of various dilutions of the antibiotic mix (ceftazidime/amikacine/meropenem) during the 22 h incubation period (Fig. S4). A 100-fold diluted antibiotic solution, in GTSF-2, was found to sufficiently limit extracellular bacterial growth, i.e. resulting in <2% extracellular bacteria compared to the bacterial population that survived/grew intracellularly. Based on these results, cultures were treated for 2 h with a combination of ceftazidime, meropenem and amikacin (1 mg/ml each), with this treatment being initiated after 2 h of infection. Subsequently, cells were washed twice with HBSS and a 1/100 dilution of the antibiotic mix was added for an additional 22 h. Using this protocol, on average 3.7 × 10^5^ CFU/ml (standard error mean (SEM): 2.2 × 10^5^) for wild type *B. cenocepacia* were recovered from the cells after 24 h, whereas only 5.2 × 10^3^ CFU/ml (SEM: 3.2 × 10^3^) were recovered from the supernatant; this low number (less than 2% of the total population) does affect the outcome of the experiment.

### 3.6 Role of selected small proteins in the infection process and cytotoxicity of lung epithelial cells

Using this optimized protocol, we first determined whether the small proteins were expressed during infection of A549 lung cells. Only small proteins for which expression was confirmed using the translational fusion reporters were included. For all reporter strains, fluorescent bacteria were observed associated with the epithelial cells, confirming expression of these small proteins during infection (Fig. S5).

**Fig. 5:**
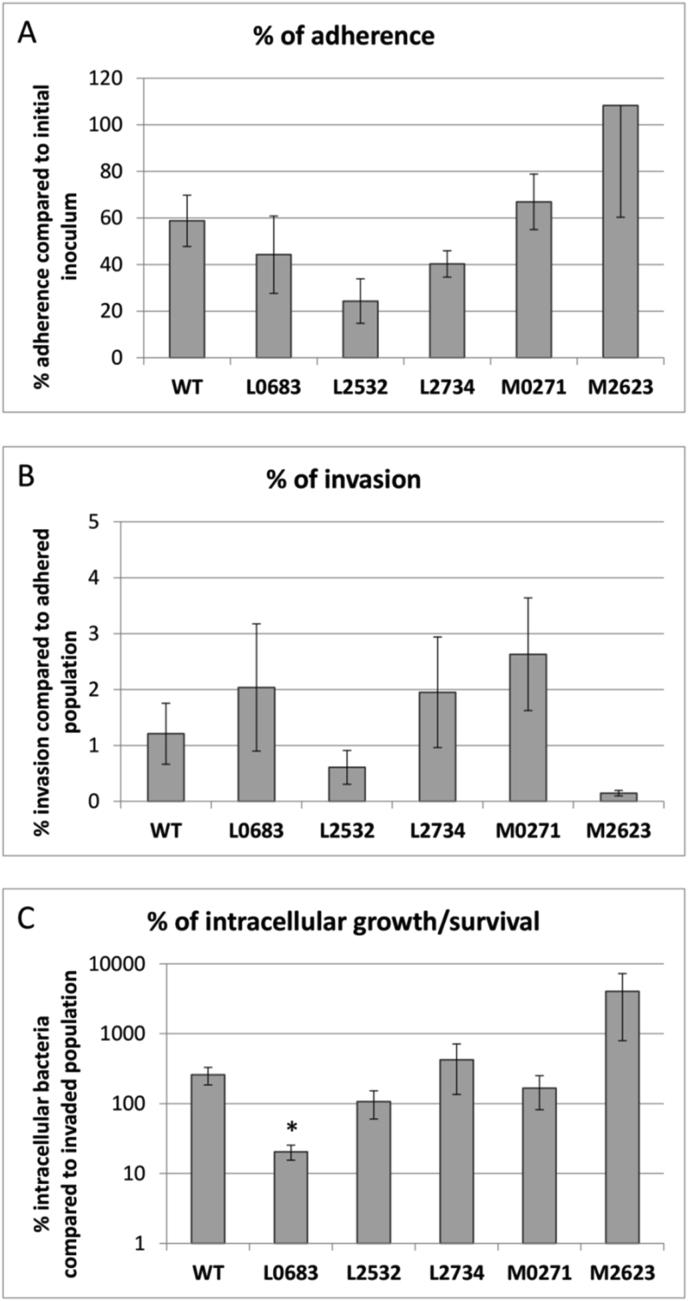
Infection of A549 lung epithelial cells with *B. cenocepacia* J2315 small protein overexpression mutants. (a) Percentage adherence of bacteria to A549 lung cells, expressed as the number of CFUs that adhered 2 h post-infection compared to the initial inoculum. (b) Percentage of invasion, expressed as the number of CFUs recovered 4 h post-infection compared to the number that adhered. (c) Percentage of intracellular growth/survival, expressed as the number of CFUs recovered 24 h post-infection compared to the fraction that invaded. Error bars represent SEM (n = 4). Significant differences are indicated with an asterisk. WT = vector control. MOI = 100:1.

Next, the role of the different small proteins in adhesion, invasion and intracellular survival was investigated. The vector control and all the mutants were able to adhere and invade the lung cells (Fig. 5). For the WT 58.8% (SEM: 11.0%) of the cells present in the inoculum were able to adhere and 1.2% (SEM: 0.6%) of the adhered bacteria also invaded the lung cells. This is similar to results obtained by Pirone et al. (2008) who found that approx. 1% of the adhered *B. cenocepacia* J2315 invaded the lung cells after 2 h of infection [38]. There were no significant differences in adherence and invasion between the overexpression mutants and the WT, but significant differences were observed in intracellular growth/survival. While for the WT and most of the overexpression mutants full survival or even growth was observed, this was not the case for the BCAL0683 overexpression mutant for which only 20.0% of the cells survived intracellularly (Fig. 5). This could be in part due to its observed slower growth and decreased biofilm formation (Fig. 2, Fig. 3). For the BCAM2623 overexpression mutant a 40.3-fold increase in the number of intracelullar CFUs was observed between 2 and 24 h whereas only a 2.5-fold increase was observed for the WT. Due to inherent variability of the assay this difference was not statistically significant, but interestingly, overexpression of this gene also increased biofilm formation (Fig. 3).

**Fig. 6:**
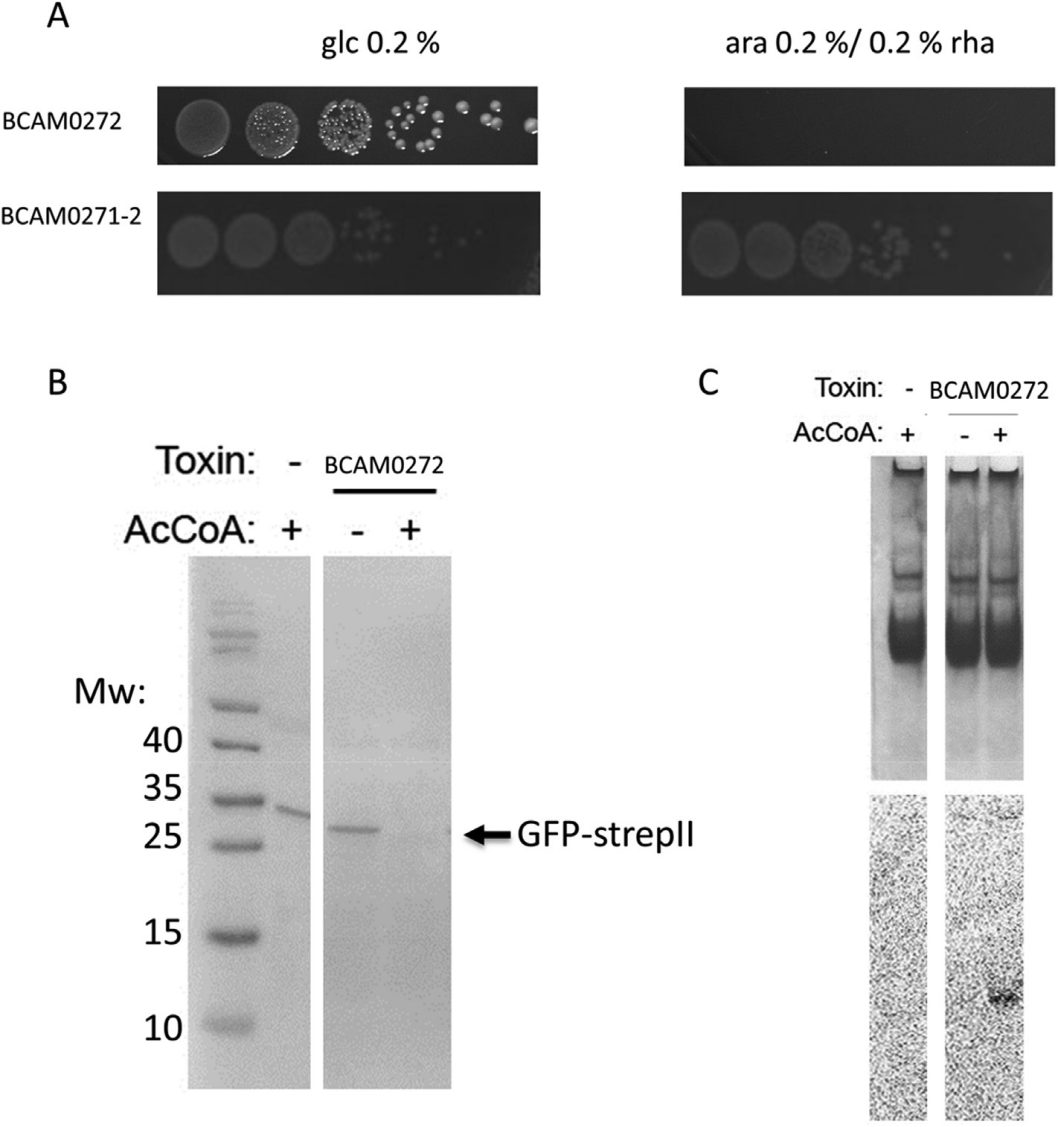
(a) The BCAM0271-2 gene pair constitutes a type II TA system. Overnight cultures of *E. coli* strains expressing BCAM0272 or BCAM0271-2 were serially diluted (10^−3^ to 10^−8^, left to right). Dilutions were spotted on LBA with repressor (glucose 0.2%) and inducer (arabinose 0.2% (w/v) or rhamnose 0.2% (w/v)). (b). Synthesis of GFP-StrepII reporter protein expressed from the T7 promoter in an *in vitro* transcription-translation system in presence of BCAM0272 with (+) and without (−) acetyl-Coenzyme A. Products of reaction resolved by SDS-PAGE and visualised by Western Blot with anti-strepII-tag antibodies. (c) Acetylation of tRNA pool in an *in vitro* transcription-translation system by different GNAT toxins with (+) and without (−) [14C]acetyl-Coenzyme A. RNAs resolved by native PAGE and stained with methylene blue (top panel), gel was then dried and exposed to phosphor storage screen (bottom panel).

Finally, cytotoxicity of the different mutants was evaluated based on a LDH assay. The release of cytosolic LDH by the lung epithelial cells following exposure to *B. cenocepacia* for 48 h was measured (Fig. S6). No statistically significant differences in cytotoxicity were observed between vector control and the mutants overexpressing the different small proteins. However, for two mutants (BCAL0683 and BCAL2532) cell death was consistently lower in all four biological replicates compared to vector control. For BCAL0683 this might be due to the slower growth and poor intracellular survival of the overexpression mutant compared to vector control (Fig. 2, Fig. 6).

### 3.7 The small protein BCAM0271 is part of a type II toxin antitoxin module

The small protein BCAM0271 is located adjacent to another small protein, BCAM0272 which was identified as a toxin belonging to a type II TA-module in a previous study [34]. Type II TA-modules are small genetic entities that usually consist of two genes: one encoding a toxin which can inhibit an important cellular function and another encoding an antitoxin which can form a complex with the toxin and inactivate it [11]. RNA-Seq coverage data suggest that BCAM0271 and BCAM0272 form one operon [19], and expression values are similar for both genes across all RNA samples investigated [17] (Fig. S7).

To confirm that BCAM0271 is part of a TA-module, we cloned BCAM0272, encoding the putative toxin, alone or in combination with BCAM0271, encoding the putative antitoxin, in vectors carrying inducible promoters and tested the effect on *E. coli* viability. Growth was significantly inhibited in the presence of the inducer when only BCAM0272 was expressed, while the presence of the inducer had no effect on growth when BCAM0271 and BCAM0272 were coexpressed, confirming that BCAM0271 can neutralize BCAM0272 toxicity (Fig. 6) and that the operon thus consists of a true TA-module.

Free toxins can impede various cellular processes like DNA replication, ATP or cell wall synthesis, but most toxins interfere with translation [39]. The most common mechanism of action of toxins relies on mRNA degradation [40], while other mechanisms include degradation of tRNA [10,41], phosphorylation of EF-Tu [42], and inhibition of glutamyl-tRNA synthetase which leads to the accumulation of uncharged tRNAglu [43]. Recently, a novel TA-family inhibiting translation was described, in which the toxins are tRNA acetyltransferases [44]. BCAM0272 possesses a Gcn5-related acetyltransferase (GNAT) domain and was predicted to encode a N-acetyltransferase, suggesting similar activity. To confirm inhibition of translation by the BCAM0272 toxin, *in vitro* translation of a reporter protein (GFP-strepII) was tested in the presence and absence of acetylcoenzyme A. Products of the reaction were resolved by SDS-PAGE and visualized using Western Blot with anti-strepII-tag antibodies. No product was observed in the presence of acetyl-CoA confirming that BCAM0272 inhibits translation in the presence of acetyl-CoA (Fig. 6). To date, AtaT, identified in *E. coli* and TacT, identified in *Salmonella* typhimurium, are the best-characterized acetyltransferase toxins [44,45]. Both were found to block translation by acetylating tRNA, but the specificities of these toxins are different. While AtaT inhibits translation initiation by N-acetylating tRNA^fMet^, TacT acetylates elongator tRNAs. To test whether BCAM0272 also targets tRNAs, a purified mixture of tRNAs from *E. coli* was treated with BCAM0272 in the presence and absence of [14C]acetyl-CoA. Autoradiography confirmed that BCAM0272 also acetylates tRNAs (Fig. 6). Based on these results we can conclude that BCAM0271-2 encodes a bona fida TA-module which targets translation by acetylating tRNAs. TA-modules are thought to be involved in several biological processes including regulation of gene expression, growth control, programmed cell death, biofilm formation, the stabilization of mobile elements, phage propagation and persistence [46]. We found that the antitoxin overexpression mutant grew slightly better in LBB compared to the vector control, but worse in acidic conditions (Fig. 2b), suggesting a role for this module in growth under stress conditions. Antitoxin BCAM0271 was also upregulated after treatment with Tob or Cip [34] and an increase in persisters was observed for the antitoxin overexpression mutant after treatment with Tob or Cip, suggesting a role in persistence. Since bactericidal antibiotics kill cells by corrupting cellular functions which are inhibited by toxins, it has been suggested that overexpression of the toxin can prevent antibiotics from killing and give rise to persister cells [47].

While the role of TA-modules in persistence has previously been documented [46], their role was recently questioned [48]. We previously found that overexpression of the toxin BCAM0272 [34] led to an increase in survival after treatment with high concentration of Tob or Cip. Interestingly, in the present study we found that overexpression of the antitoxin BCAM0271 similarly increased survival. The link between toxins and persistence, with more toxin production leading to increased persistence may thus not always be as straightforward as previously thought.

## 4 Conclusion

A large number of small proteins are present in the genome of *B. cenocepacia* J2315. The function of many of them is still unknown, but our data suggest that at least some of them are expressed and involved in important biological processes like growth, biofilm formation, persistence, and intracellular survival. We used two different approaches to investigate production of small proteins, an LC-MS based proteomics approach and construction of translational eGFP fusion reporters. Both approaches allowed to identify expressed small proteins, but the overlap between small proteins identified with both approaches was limited, highlighting the importance of using different techniques. One of the small proteins identified in this study, BCAM0271, is the antitoxin in a TA-module which targets translation by acetylating tRNAs.

## Conflicts of interest

The authors declare that there are no conflicts of interest.

## Funding information

This research was financially supported by the Research Foundation Flanders (Postdoctoral fellowship to HVA, Odysseus grant to AC) and the Belgian Science Policy Office (Interuniversity Attraction Pole Program) (TC, LVM).

## Appendix A Supplementary data

Supp1 Supp1

Supp2 Supp2

Supp3 Supp3
