# Supplementary material for: The role of small proteins in Burkholderia cenocepacia J2315 biofilm formation, persistence and intracellular growth
Source: Biofilm. 2019 Jun 15;1:100001. doi: 10.1016/j.bioflm.2019.100001 (PMC7798474; doi:10.1016/j.bioflm.2019.100001)

Table S1. Primers used in the study.

Gene	Forward	Reverse	Annealing temp (°C)
For construction of eGFP translational fusion reporters			
BCAL0008a	ATTATAACATATGGCAGCGTCATTCCGTCGATG	ATAAGATCTGAGTTTGAGTTCGTCGCCGCTC	60
BCAL0683	ATACAACATATGGCTCCGGCACCCGATGCAT	CGCAGATCTGTTCTTGTCGACGATTTCGTATC	65
BCAL2532	ATTATAACATATGCGAGACCTACCTGTCCGAC	TATAGATCTGGCCTTGCGCAGTTGGTCATGC	60
BCAL2734	GTATCACATATGCCGCTGCATCGACGATCC	TATAGATCTGTTGCCGAACGTCGCTTCCCA	60
BCAM0271	GTTATAACATATG**C**GCCGTGCCGACATCTCTTG	TATAGATCTCGCCGCGTGCACATCCGTTTC	60
BCAM2623	CGTATAACATATGGAACCAGGTCAACGGTATC	ATAAGATCTCGAGTGTTGGCACGTCAGAATC	60
Plasmid insert	CGTAGAGGATC TGCTCATGTT TGAC	GACGTAAACG GCCACAAGTTCA	55

Table S1. Primers used in the study (continued).

For construction of overexpression mutants			
BCAL0008a	GTGCTGCATATGAGCGAACGATTAGATCCAATC	TATCTAGATCAGCTTTCCAAGCCATCCTTAAGG	60
BCAL0683	GTACAACATATGCAAATGATCTACAACAGCCCCAAC	ATTCTAGATCAGTGCAGCACGACGGGCATC	60
BCAL2532	TTACAACATATGGGCATCGTGAACATCGACGA	ATTCTAGATCATGACGCCCCCATCTTGATG	60
BCAL2734	GTAACTCATATGAAGGGATTTCGCTTTGGTTC	ATTCTAGA TCAGACGTGGACGATTTCCC	60*
BCAL3186	GTACTACATATGACCTGTGCACGCTGATACGAC	ATTCTAGAAGTCTCGTCGATCACGCGCTC	58*
BCAM0271	GTACTACATATGGCTACCACACGATTTGAGG	ATTCTAGATCATGCGTTACGCAGCAGTTG	60
BCAM0971	GTACTACATATGAGCGATTCGCATCAATCCGAC	ATTCTAGA GGCACAGCGCGTTACACCTTG	60
BCAM2623	GTGCCGCATATGATGATCGAAGATACCGTTTTC	TATCTAGATCAGAACTGCAGCCGGCCGT	60
pBCA050	GTGCTGCATATGGGGATCACTCAGGAAGAGCTA	TATCTAGATCAGTCCTGCGACAAACTCACCAGC	60
Plasmid insert	CACGTTCATCTTTCCCTGGT	GCTGTTTTGGCGGAGTGAG	58

For construction of plasmids containing BCAM0272 or BCAM0271-2			
BCAM0272	GATCTCTAGAAGGAGGGAGTAATGAGCGGTGCGCAGTTGG	GACTCTGCAGCTACTTCACCGTTGCCAATGGC	
BCAM0271-2	GTACTACATATGGCTACCACACGATTTGAGG	CGTCTAGACTACTTCACCGTTGCCAATGGCATG	

Restriction sites are underlined. * A different kit and cycling conditions were used (Hotstar Hifidelity Qiagen, cycling conditions: 5 min 95°C, 30 times 15s 94°C, 1min 60°C, 25s 72°C and finally 10min at 72°C)

Table S2. Overview of all *B. cenocepacia* J2315 genes encoding small protein < 100 amino acids.

(a)All small proteins

(b) Small proteins with a known function

(c) Small proteins annotated as hypothetical for which similarity could be found with known genes in other bacteria.

(d) Small proteins annotated as hypothetical without similarity with known genes in other bacteria. Fold change in expression in different conditions is also presented. Numbers refer to expression levels after treatment compared to expression in untreated cultures (or to expression in biofilm vs. expression in planktonic cells). Tob: tobramycin, CHX: chlorhexidine, Low Fe: low iron, BF: biofilm, PL: Planktonic growth

See separate Excel file (TableS2.xls)

Table S3. Summary of the properties of all annotated small proteins.

See separate Excel file (TableS3.xls)

Figure S3. Effect of overexpression of small proteins on the growth of *B. cenocepacia* J2315. Strains were grown in media supplemented with 0.2 % rhamnose. Eight different media were tested: LBB, 1/10 diluted LBB, LBB set to pH 8.2 or 4.2, and LBB with 0.25 mM 2,2’-bipyridyl, 0.045 % (w/v) NaOCl, 0.015 % (w/v) SDS or 1.5 % (w/v) NaCl.

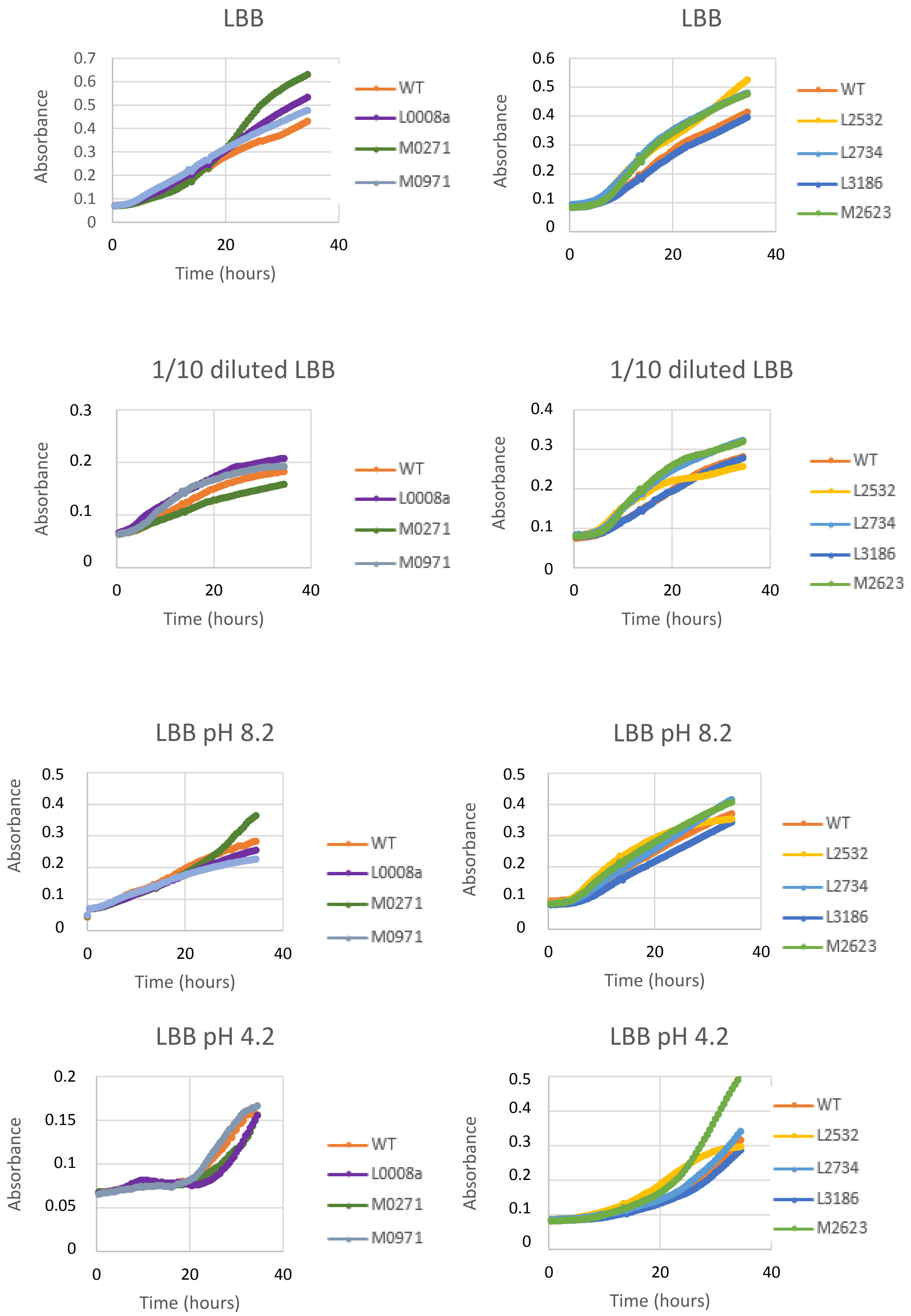

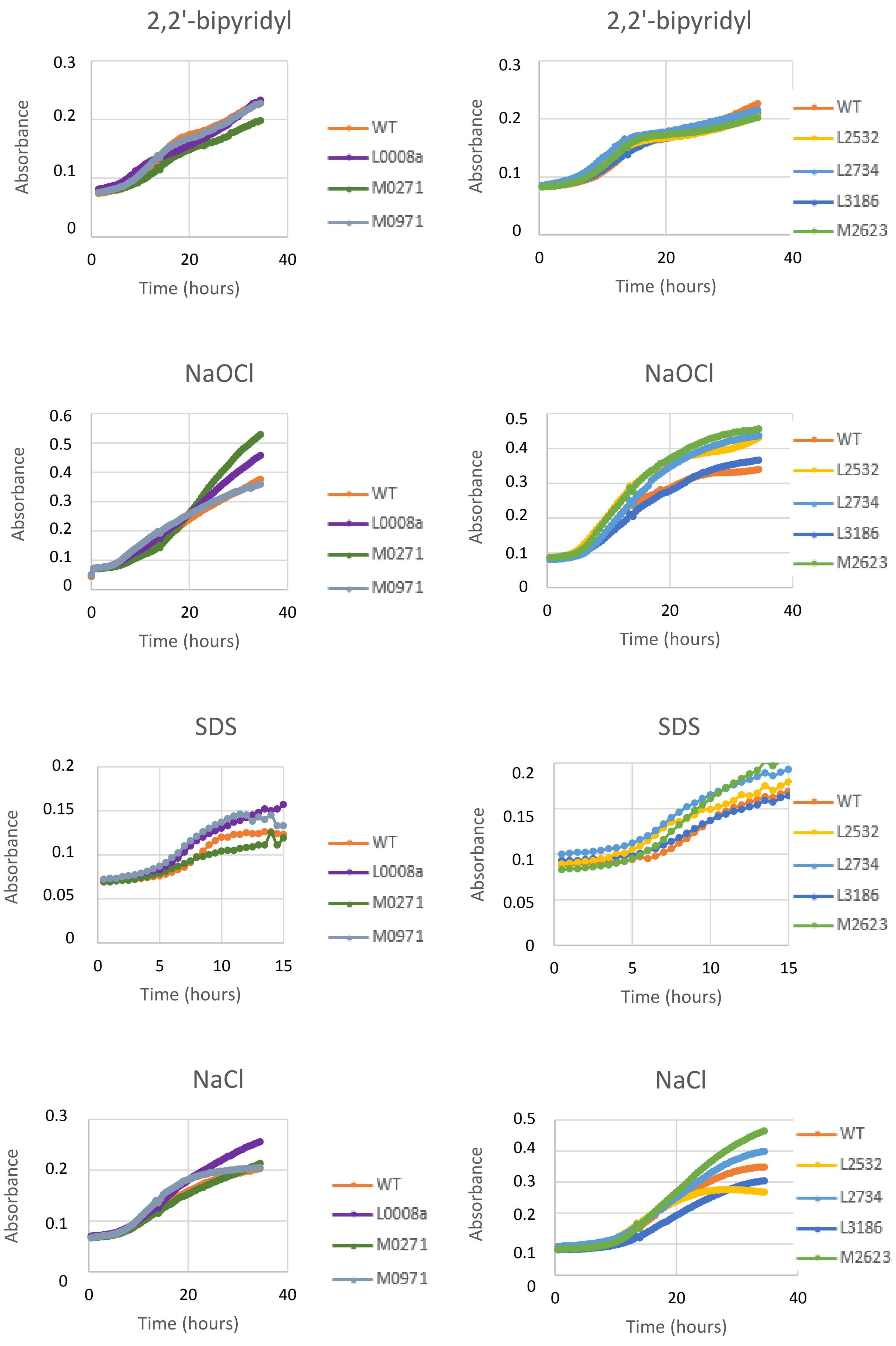
2,2'-bipyridyl
Absorbance
Time (hours)
WT
L0008a
M0271
M0971
2,2'-bipyridyl
Absorbance
Time (hours)
WT
L2532
L2734
L3186
M2623
NaOCl
Absorbance
Time (hours)
WT
L0008a
M0271
M0971
NaOCl
Absorbance
Time (hours)
WT
L2532
L2734
L3186
M2623
SDS
Absorbance
Time (hours)
WT
L0008a
M0271
M0971
SDS
Absorbance
Time (hours)
WT
L2532
L2734
L3186
M2623
NaCl
Absorbance
Time (hours)
WT
L0008a
M0271
M0971
NaCl
Absorbance
Time (hours)
WT
L2532
L2734
L3186
M2623

Figure S4. (a) Microscopic image of A549 cells infected for 2 h with a *B. cenocepacia* J2315 rhamnose inducible eGFP expressing mutant (MOI: 100:1). After infection cells were treated for 2 h with an antibiotic mix (amikacin, meropenem and ceftazidime; 1 mg ml^{-1} each) and incubated for 22 h in the absence or presence of different dilutions (1/100, 1/200, 1/400, 1/800) of this antibiotic mix. (b) Number of CFUs recovered from the supernatant of infected epithelial cells exposed to different dilutions of the antibiotics. Error bars represent the standard deviation (n = 3 technical repeats).

(a)

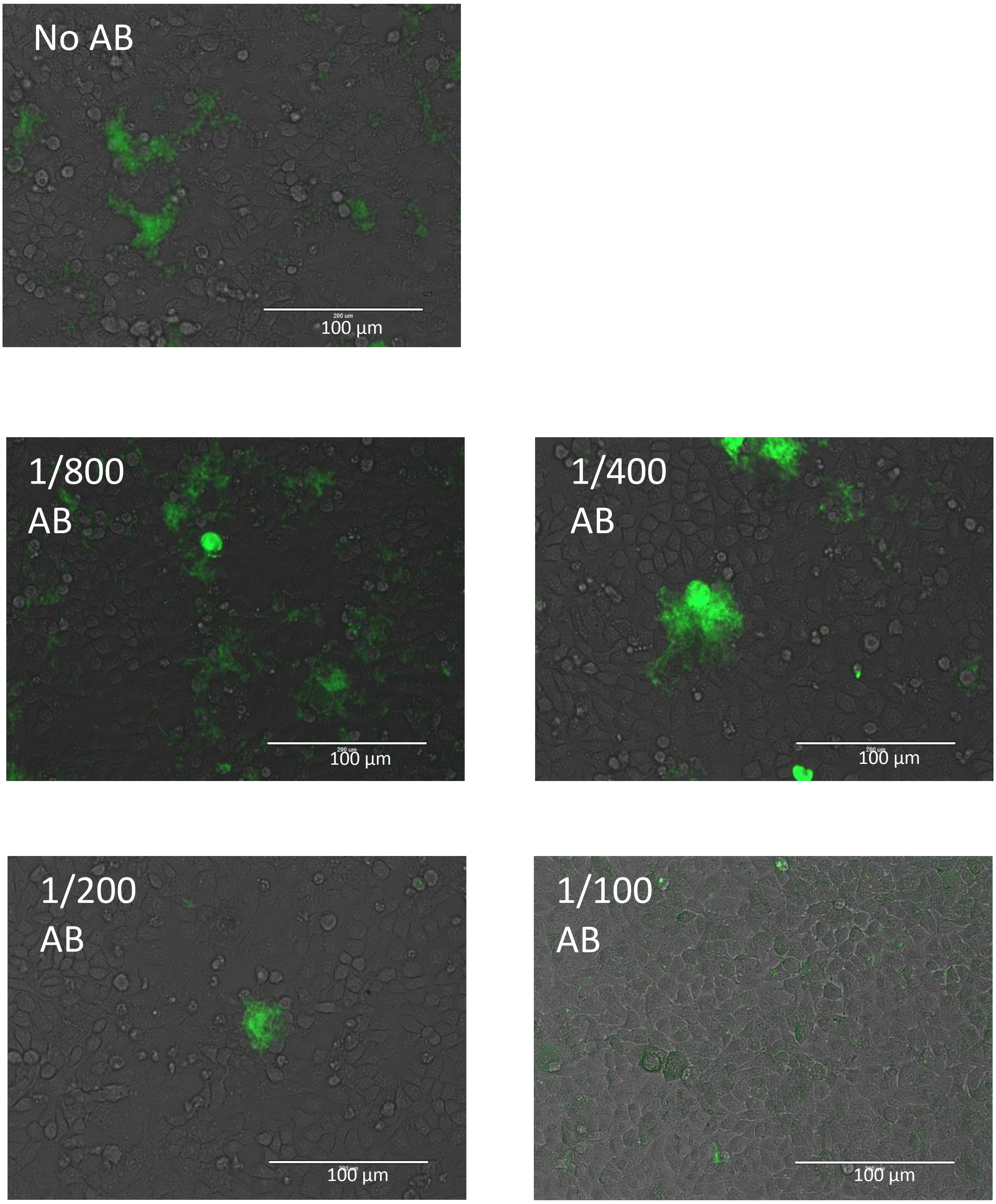

(b)

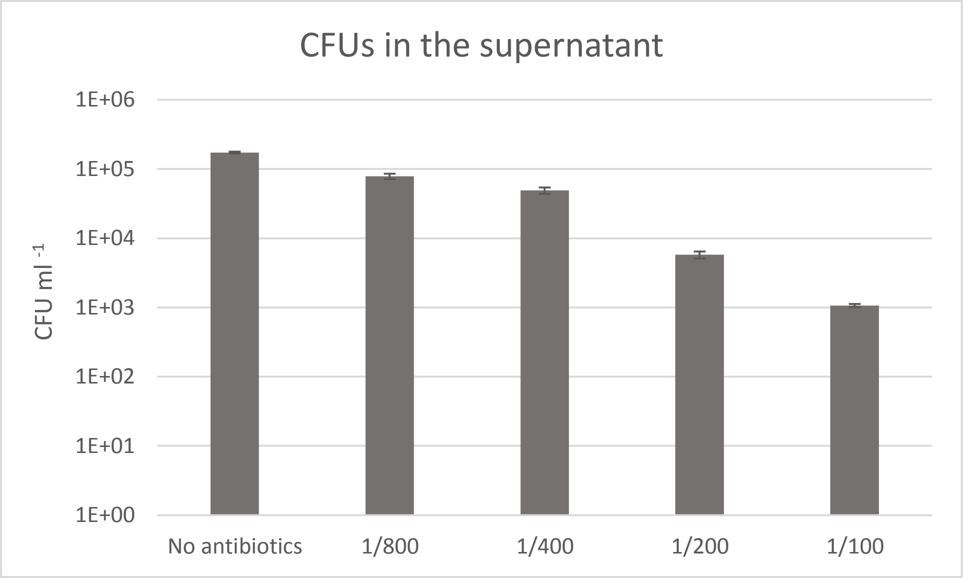
CFUs in the supernatant
1E+06
1E+05
1E+04
1E+03
1E+02
1E+01
1E+00
CFU ml -1
No antibiotics
1/800
1/400
1/200
1/100

Figure S5. Microscopic images of A549 cells infected with *B. cenocepacia* J2315 eGFP translational fusion reporters. An overlay of light microscopic and fluorescence images is presented. Positive control= *B. cenocepacia* J2315 with plasmid pScRhaB2 containing eGFP under a rhamnose inducible promotor.

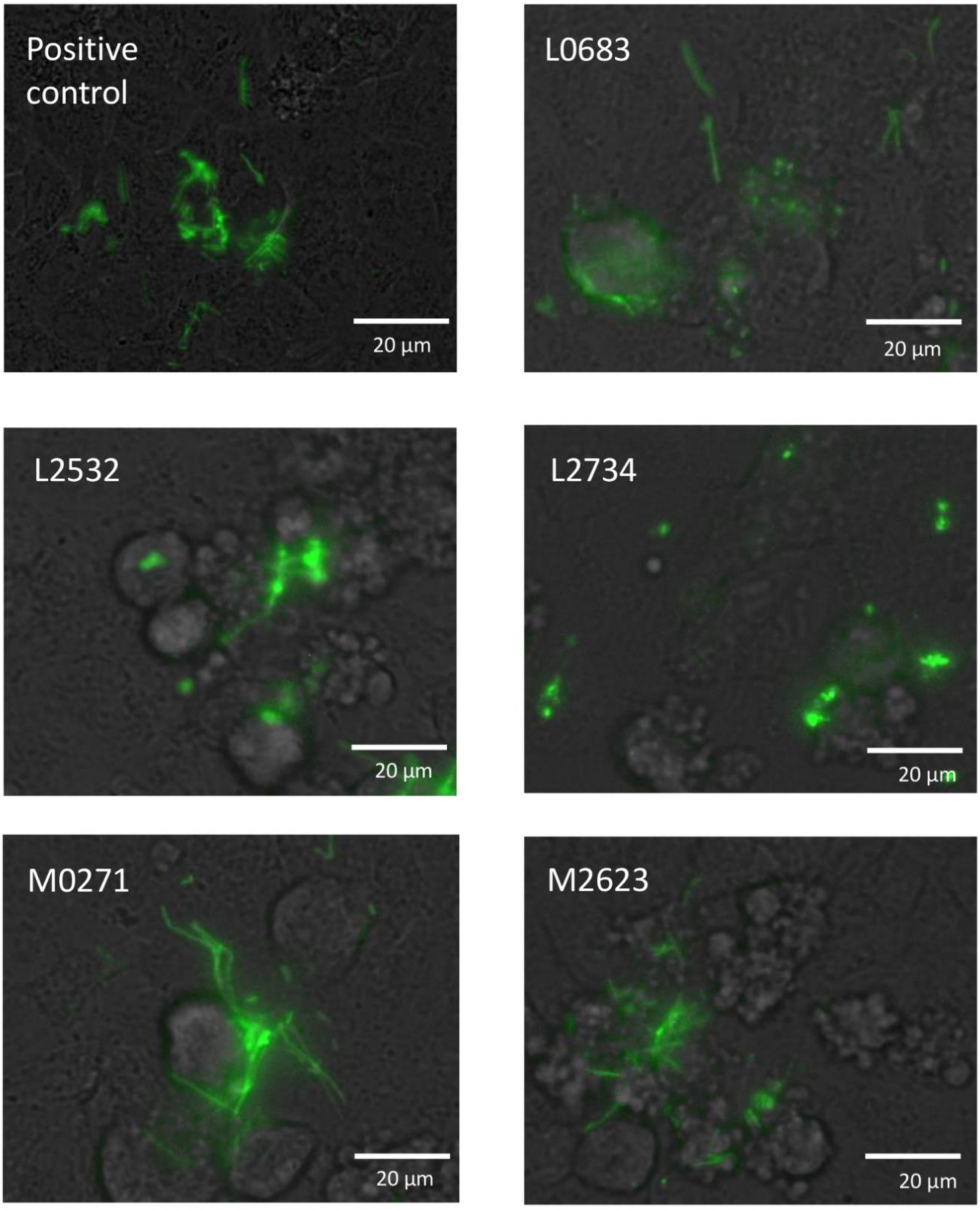

Figure S6. Lactate dehydrogenase (LDH) release of A549 lung epithelial cells that were non-infected (=blank) and infected for 48h with different *B. cenocepacia* J2315 small protein overexpression mutants or the vector control (MOI: 100:1). % cell death is presented as a percentage of a positive control (= lung epithelial cells lysed with Triton-X100) set to 100 % cell death. Data are average of 3 experiments, error bars represent standard deviation.

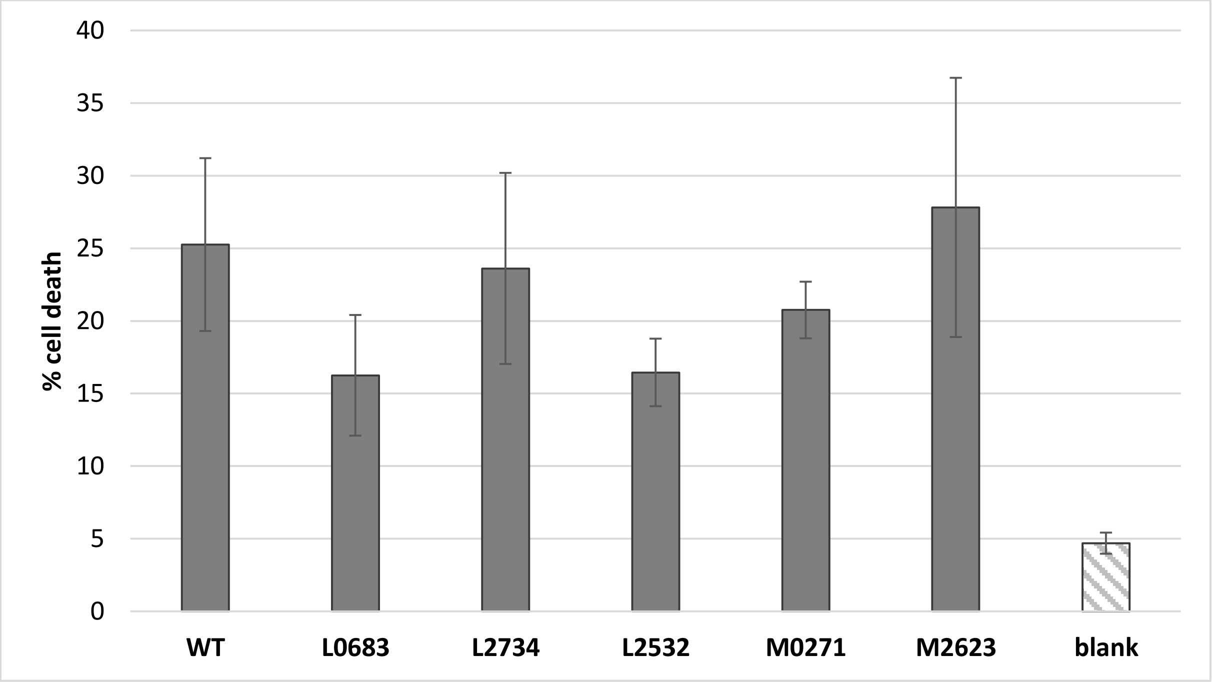

Figure S7. Genetic organisation of the BCAM0271-2 operon.
Top panel: Differential RNA sequencing (dRNA-Seq) not treated with Terminator 5´-phosphate-dependent exonuclease (TEX).
Middle panel: dRNA-Seq with TEX treatment. native RNA species carrying a triphospate at the 5'end, as can be found at TSS, are not degraded, leading to relative enrichment of reads at TSS. dRNA-Seq is a 5'end sequencing method carried out without size fractionation. This retains short RNA species, and leads to over-representation of the 5'end of RNAs, while the 3'end of longer transcripts is mostly not covered.
Bottom panel: Conventional RNA sequencing ("global" gRNA-Seq), where short RNA species are lost during library preparation, but longer RNAs are represented by full coverage. The transcript for BCAM0271 and BCAM0272 appears uninterrupted, suggesting these genes are transcribed as one operon. The 5'UTR of this operon is probably cleaved and then lost during library prep. All RNA samples are derived from *B. cenocepacia* J2315 biofilms grown in microtiter plates.
The numbers below the genes indicates the RPKM (average ± standard deviation, n=6). RPKM values for BCAM0271 and BCAM0272 are not significantly different; while the values for BCAM0273 are significanty lower ($p < 0.001$).

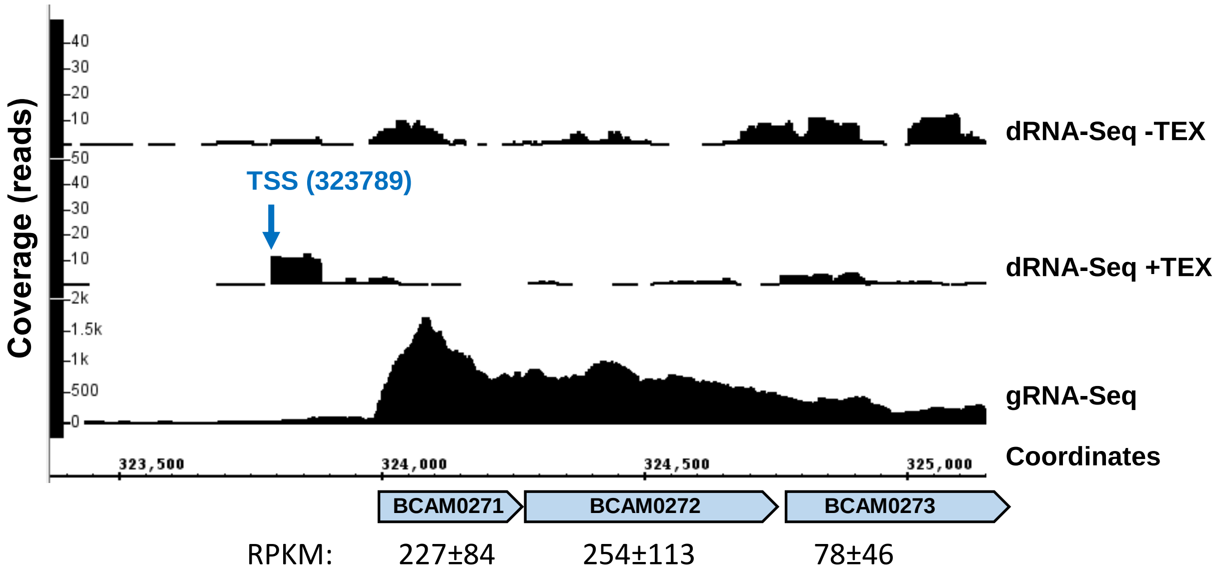

Supplement: Supp1 [file mmc1.pdf]
